# Incorporating a Stepped Care Approach Into Internet-Based Cognitive Behavioral Therapy for Depression: Randomized Controlled Trial

**DOI:** 10.2196/51704

**Published:** 2024-02-09

**Authors:** Jasleen Kaur Jagayat, Anchan Kumar, Yijia Shao, Amrita Pannu, Charmy Patel, Amirhossein Shirazi, Mohsen Omrani, Nazanin Alavi

**Affiliations:** 1 Centre for Neuroscience Studies Queen's University Kingston, ON Canada; 2 Department of Psychiatry Queen's University Kingston, ON Canada; 3 Pastoral Studies, Toronto School of Theology (TST) Emmanuel College University of Toronto Toronto, ON Canada; 4 Online Psychotherapy Tool Toronto, ON Canada

**Keywords:** internet-based cognitive behavioral therapy, i-CBT, major depressive disorder, MDD, stepped care, digital mental health care, mobile phone

## Abstract

**Background:**

Depression is a hidden burden, yet it is a leading cause of disability worldwide. Despite the adverse effects of depression, fewer than one-third of patients receive care. Internet-based cognitive behavioral therapy (i-CBT) is an effective treatment for depression, and combining i-CBT with supervised care could make the therapy scalable and effective. A stepped care model is a framework for beginning treatment with an effective and low-intensity intervention while adapting care based on the patient’s needs.

**Objective:**

This study investigated the efficacy of a stepped care i-CBT model for depression based on changes in self-reported depressive symptoms.

**Methods:**

In this single-blinded, randomized controlled trial, participants were allocated to either the i-CBT–only group (28/56, 50%) or the i-CBT with stepped care group (28/56, 50%). Both groups received a 13-week i-CBT program tailored for depression. The i-CBT program was provided through a secure, online mental health clinic called the Online Psychotherapy Tool. Participants read through the sessions and completed the assignments related to each session. Participants in the stepped care group received additional interventions from their care provider based on standard questionnaire scores (ie, Patient Health Questionnaire–9 [PHQ-9], Quick Inventory of Depressive Symptomatology [QIDS], and Quality of Life Enjoyment and Satisfaction Questionnaire–Short Form) and their assignment responses. From lowest to highest intensity, the additional interventions included SMS text messages, phone calls, video calls, or a video call with a psychiatrist.

**Results:**

For this study, 56 participants were recruited to complete an i-CBT program (n=28, 50%; mean age 37.9; SD 13.08 y; 7/28, 27% were men) or an i-CBT with stepped care program (n=28, 50%; mean age 40.6; SD 14.28 y; 11/28, 42% were men). The results of this study indicate that the i-CBT program was effective in significantly reducing depressive symptoms, as measured by the PHQ-9 (*F*_4,80_=9.95; *P*<.001) and QIDS (*F*_2,28_=5.73; *P*=.008); however, there were no significant differences in the reduction of depressive symptoms between the 2 groups (PHQ-9: *F*_4,80_=0.43; *P*=.78; QIDS: *F*_2,28_=3.05; *P*=.06). The stepped care group was not significantly better in reducing depressive symptoms than the i-CBT group (PHQ-9, *P*=.79; QIDS, *P*=.06). Although there were no significant differences observed between the number of participants who completed the program between the groups (*χ*^2^_1_=2.6; *P*=.10), participants in the stepped care group, on average, participated in more sessions than those who prematurely terminated participation in the i-CBT group (*t*_55_=−2; *P*=.03; 95% CI –4.83 to –0.002).

**Conclusions:**

Implementing a stepped care approach in i-CBT is an effective treatment for depression, and the stepped care model can assist patients to complete more sessions in their treatment.

**Trial Registration:**

Clinicaltrials.gov NCT04747873; https://clinicaltrials.gov/study/NCT04747873

## Introduction

### Background and Rationale

Depression is a leading cause of disability, affecting approximately 3.8% of the population worldwide [1,2]. Major depressive disorder (MDD) is characterized by persistent feelings of sadness, negative mood, or loss of interest in life activities [3]. Detrimental and persistent changes in appetite, sleep, energy, and cognition may accompany these feelings. In addition to its deleterious impacts on mental health, depression is associated with increased morbidity, decreased quality of life, and reduced work productivity [4-6]. Despite the negative consequences of depression, only one-third of individuals receive treatment, and of those, only 3 in 5 people receive sufficient care [7,8].

Cognitive behavioral therapy (CBT) is an effective treatment for MDD [9-11]. It is a form of psychotherapy that focuses on cognitive restructuring strategies and behavioral activation techniques to help individuals with depression overcome their depressive symptoms and modify ineffective thinking patterns. Although effective, CBT on its own presents some challenges such as accessibility issues, lack of follow-ups, and increased costs [12-14]. Over the past 2 decades, issues of accessibility are being increasingly addressed through digital modalities, such as internet-based CBT (i-CBT) [11,15,16]. i-CBT allows for the delivery of CBT through digital media including computers and smartphones. It involves using web-based sessions with interactive components and practices to teach individuals ways to improve their mental health. Many studies have shown the efficacy of i-CBT for depression and anxiety, with results comparable with those of traditional in-person CBT [11,16-20]. The digital format allows for increased accessibility and convenience when delivering and receiving psychotherapy while remaining effective [21,22]; however, the digital format comes with limitations, including low adherence. Adherence rates are variable and can be affected by multiple factors including the digital modalities used and patient-therapist interactions [23-25]. Furthermore, attrition rates in CBT are the highest in patients with depression [26], and adherence can be compromised as disease severity increases [27]. With the benefit of being versatile and adaptable, i-CBT may be adapted to include support to address the issues of adherence.

The stepped care model is an approach to providing individuals with adaptable and effective care that is adjusted based on symptom severity [28-31]. This dynamic approach allows care to be adapted as required throughout an individual’s treatment plan to best tailor care to the individual’s needs. Stepped care models aim to provide the most effective and efficient care by minimizing resources and costs for care. The least intensive intervention, such as self-help materials or psychoeducation, is first provided to patients in a stepped care model. The patient may then advance to the next level of care, which may include more extensive interventions such as increased support or medication if they do not improve or just partially improve. Until the patient gets the desired result or reaches the highest level of care, this process is repeated. This stepped care model allows for efficient allocation of resources, increases access to care, can reduce wait times, can lower costs, and can increase versatility in treatment options [28,31]. This model is effective in treating mental health disorders including MDD and focuses on a patient-centered approach to care [21,31].

In the stepped care model, all patients receive some form of treatment for their symptoms, and based on the symptom severity and progress through treatment, additional care is added to their treatment plan. The addition of therapist-guided support to i-CBT has demonstrated high levels of patient satisfaction and a general decrease in symptom severity [32]. It is also noted that dropout rates decrease as increased therapeutic support is provided to patients. A meta-analysis that gathered dropout rates of CBT based on treatment support levels found that without support, the dropout rate was 74%; with administrative support, the dropout rate was 38%; and with therapeutic support, the patient dropout rate was 28% [16]. This suggests that the type of support provided to patients is important. Therapist-guided support in an i-CBT setting can also include text (ie, messaging and emails) or audio-based (ie, telephone and video) formats, and the mode of therapy may influence treatment outcome [22,33,34]. Incorporating text and call interventions into online therapy can provide an additional support outlet for patients throughout their treatment by providing an additional means of communication while remaining accessible [35]. Phone and video interventions have both been shown to be effective in reducing symptoms and maintaining patients in care compared with in-person care for mood disorders [36]. Phone calls may be more broadly accessible than video calls owing to inherent technological barriers; however, video calls allow for additional nonverbal cues to be assessed in care [36]. They can serve as check-ins, encouragement, and reminders and help monitor patient progress between treatment sessions. Through this additional support, a review of therapeutic concepts and words of encouragement are found to help decrease symptom severity and improve therapy completion [37]. This method can help with building therapist-patient rapport throughout treatment and act as a valuable tool in online therapy.

### Objectives

This study was a single-blinded, randomized controlled trial exploring the efficacy of a stepped care model in an i-CBT program compared with an i-CBT–only program for adults with MDD. The i-CBT program for depression was provided through a secure, online mental health clinic called the Online Psychotherapy Tool (OPTT) [38]. The stepped care model considered the varying digital modalities of text-based, phone-based, and video-based interventions to help support individuals in their care.

## Methods

### Ethical Considerations

The research study was approved by the Health Sciences and Affiliated Teaching Hospitals Research Ethics Board (file number 6031992) at Queen’s University in Kingston, Ontario, Canada. The research study followed the CONSORT-EHEALTH (Consolidated Standards of Reporting Trials of Electronic and Mobile Health Applications and Online Telehealth) guidelines and can be viewed in Multimedia Appendix 1 [39].

No monetary compensation was provided to participants for participation in this study because they were provided with CBT treatment for their symptoms.

### Data Privacy

To maintain participant confidentiality, each participant was assigned a randomized participant ID number. This ID was used to identify the participant for data analysis purposes. Only associated care providers had access to the participant’s identity owing to the nature of the CBT intervention. If any technical issues on OPTT arose, this ID was used to resolve the issues to maintain participant anonymity.

Participant information maintained online was password protected, including consent forms, identities, and CBT content. All encrypted files were stored on a safe server run by Queen’s University. Participant identities and consent forms are stored on-site at Queen’s University’s exclusive storage in Kingston, Ontario, Canada, for 5 years following the conclusion of the study because they are considered as medical records. Following the 5 years, the participant records will be destroyed. Participants were informed about the possibility to withdraw from the study at any time if they wished to do so. In upcoming plans for knowledge dissemination and publication of outcomes, participant identification will be secured and maintained anonymously.

The online platform used to deliver the i-CBT program, OPTT [38,40], serves as the repository for all data. OPTT complies with the Personal Information Protection and Electronic Documents Act, Health Insurance Portability and Accountability Act, and Service Organization Control–2. The cloud infrastructure of Amazon Web Service Canada is used to host all servers and databases, and Medstack manages it to ensure compliance with all the local, state, and federal privacy and security laws. For privacy reasons, OPTT does not gather any identifying personal data or IP addresses. Only anonymous metadata were gathered by OPTT to enhance the quality of its services and provide the care provider team access to participant analytics (ie, interaction with the OPTT platform). No OPTT employee had direct access to participant data owing to data encryption procedures (ie, participant ID). All encrypted backups are stored at Queen’s University.

### Outcome Evaluation

#### Patient Health Questionnaire–9

The Patient Health Questionnaire–9 (PHQ-9) is a self-assessment questionnaire designed to examine a participant’s depression severity through a 9-item questionnaire, with an additional question about functional health [41]. Each of the 9 items is scored from 0 (not at all) to 3 (nearly every day) and corresponds to the *Diagnostic and Statistical Manual of Mental Disorders, Fifth Edition* criteria for MDD symptoms [3]. Each item is then scored for a total to indicate depression severity from none to severe, ranging from 0 to 27. High scores indicate high depression.

#### Quick Inventory of Depressive Symptomatology Questionnaire

The Quick Inventory of Depressive Symptomatology (QIDS) questionnaire is a self-assessment questionnaire designed to screen for depression and measure depression severity based on 16 items [42]. Each item correlates with the *Diagnostic and Statistical Manual of Mental Disorders, Fifth Edition* criteria for MDD symptoms and is scored using a 4-point scale (0-3), for a total score ranging between 0 and 27 [3]. To calculate the final score, the questions are summed using the highest response in the following domains: sleep patterns (questions 1-4), sad mood (questions 5), change in appetite (questions 6-9), concentration and decision-making (questions 10), self-view (questions 11), suicidal thoughts (questions 12), general interest (questions 13), energy level (questions 14), and psychomotor effects (questions 15 and 16). High scores indicate high depression severity.

#### Quality of Life Enjoyment and Satisfaction Questionnaire–Short Form

The Quality of Life Enjoyment and Satisfaction Questionnaire–Short Form (Q-LES-Q) is a self-assessment questionnaire designed to collect information about the level of enjoyment and satisfaction in various aspects of daily functioning through a series of 16 items [43]. Each item is rated on a scale from 1 (very poor) to 5 (very good). Of the 16 items, only the first 14 items are summed for a total raw score ranging from 14 to 70. High scores indicate great quality of life.

### Measured Outcomes

The primary outcomes measured in this study included changes in depressive symptoms, as measured by the PHQ-9 and QIDS. Using the Q-LES-Q to measure changes in the participant’s quality of life was also a primary outcome that was evaluated. Participants completed the PHQ-9 every 3 weeks (weeks 1, 4, 7, 10, and 13) and the QIDS and Q-LES-Q at 3 time points (weeks 1, 7, and 13) throughout the program. All the 3 questionnaires are intended to be completed at the 3-, 6-, 9-, and 12-month follow-up periods during the 1-year follow-up phase (Textbox 1). Pretreatment and posttreatment changes in depressive symptoms (ie, PHQ-9 and QIDS) and quality of life (Q-LES-Q) between the control and experimental groups were compared. The number of sessions completed by each participant was a secondary measure that was considered when evaluating compliance with the i-CBT program between the 2 groups.

**Schedule of the assigned clinical questionnaires sent to participants, including the 1-year follow-up period.**

**Session 1**

- Patient Health Questionnaire–9 (PHQ-9), Quality of Life Enjoyment and Satisfaction Questionnaire–Short Form (QIDS), Quick Inventory of Depressive Symptomatology (Q-LES-Q), and demographic survey

**Session 2**

- No questionnaires are provided during these sessions.

**Session 3**

- No questionnaires are provided during these sessions.

**Session 4**

- PHQ-9 and initial stepped care assessment (if the participant is in the stepped care group, an intervention decision will be made at the end of every session, starting at session 4. Interventions will be implemented, beginning from session 5)

**Session 5**

- Interventions begin as needed (if the participant is in the stepped care group, an intervention decision will be made at the end of every session, starting at session 4; Interventions will be implemented, beginning from session 5)

**Session 6**

- No questionnaires are provided during these sessions.

**Session 7**

- PHQ-9, QIDS, and Q-LES-Q

**Session 8**

- No questionnaires are provided during these sessions.

**Session 9**

- No questionnaires are provided during these sessions.

**Session 10**

- PHQ-9

**Session 11**

- No questionnaires are provided during these sessions.

**Session 12**

- No questionnaires are provided during these sessions.

**Session 13**

- PHQ-9, QIDS, and Q-LES-Q

**Follow-up 1**

- PHQ-9, QIDS, and Q-LES-Q

**Follow-up 2**

- PHQ-9, QIDS, and Q-LES-Q

**Follow-up 3**

- PHQ-9, QIDS, and Q-LES-Q

**Follow-up 4**

- PHQ-9, QIDS, and Q-LES-Q

### Participants

This study was registered at ClinicalTrial.gov (NCT04747873). Participants were recruited from the Providence Care outpatient psychiatry clinic and Kingston Health Sciences Center sites (Hotel Dieu Hospital and Kingston General Hospital), both of which are located in Kingston, Ontario, Canada. Physicians familiar with the Queen’s Online Psychotherapy Lab (QUOPL) research team were also informed about the study and directed patients who may benefit from CBT toward the study when considered appropriate. Self-referrals were also accepted for this study. Recruitment was managed by the laboratory manager who was the initial point of contact for all participants.

### Eligibility Criteria

Individuals interested in the research study were provided with a letter of information and consent form to understand the study design before beginning the eligibility process. Following the informed consent process (written or verbal), a trained research assistant at QUOPL screened the individual based on the eligibility criteria. Individuals were eligible for the study if they met the following criteria: (1) aged ≥18 years, (2) met the criteria of MDD by a trained research assistant according to the Mini International Neuropsychiatric Interview (MINI) [44], (3) showed competence to consent to participate, (4) fluent in English because the i-CBT program was provided in English only, and (5) had consistent and reliable access to the internet. For the MINI assessment, individuals were first assessed by a trained research assistant to support a diagnosis of MDD. This MINI assessment was completed through a secure Microsoft Teams video call. Participants were ineligible for the research study if they presented with active psychosis, acute mania, severe alcohol or substance use disorder, or active suicidal or homicidal ideation. If a participant received or was receiving CBT in the past year at the time of beginning the study, they were excluded from the study to avoid confounding effects on the efficacy of this i-CBT program.

Following the consent and eligibility screening process, participants were randomized in a 1:1 allocation ratio to either the i-CBT group (control group) or the i-CBT with stepped care group (experimental group). Randomization was computer generated in Microsoft Excel using the *RANDBETWEEN* function. This method returned an integer at random that indicated either 1 (designated as the i-CBT group) or 2 (for the stepped care group). This integer was used to determine the group allocation for the participant.

**Figure 1:**
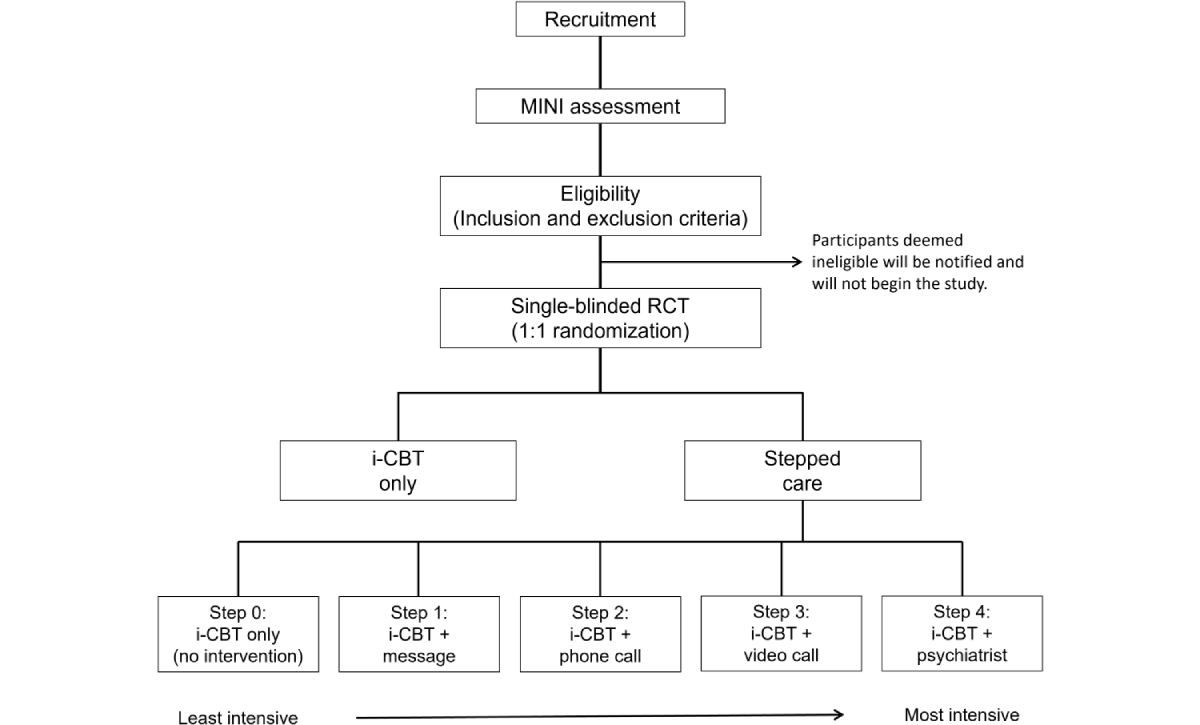
A flowchart providing an overview of the study design. i-CBT: internet-based cognitive behavioral therapy; MINI: Mini International Neuropsychiatric Interview; RCT: randomized controlled trial.

Using G*Power (version 3.1.9.7 [45]), a priori power analysis was performed to determine the minimum sample size required to test the study hypothesis. The average PHQ-9 score decreased from 16.2 before i-CBT to 11.48 (combined SD 5.45) after 12 sessions in our previous clinical trials and data collection regarding i-CBT for depression [46]. These figures led to an effect size (Hedge g of 0.86). A paired sample *t* test (1-tailed) would require 14 individuals to detect a significant impact, given the effect size and a power of 0.8. For online CBT programs, compliance and care adherence are common challenges; therefore, we predict 50% dropout rate based on previous clinical trials conducted in our laboratory [47,48]. Consequently, considering the proposed sample size calculation accounted for this dropout rate, we aimed to recruit 28 participants in each group. With the 2 treatment arms, our total sample size was expected to be 56 participants. Figure 1 summarizes the study design in a flow chart.

### Care Providers

Participants were assigned a primary care provider to help build rapport through assigning sessions, providing feedback, and providing additional interventions as needed. All care providers were trained in psychotherapy by the lead psychiatrist who is a licensed psychotherapist and expert in internet-based psychotherapy. Each participant was assigned a care team that consisted of a care provider and a psychiatrist with extensive experience in i-CBT. Care providers were research assistants recruited and trained by the team psychiatrist. Their role was to provide participants with feedback about weekly homework and act as a point of contact for any questions that the participants may have regarding the study design. The care providers included master’s degree students studying in the field of neuroscience and psychology, associated with QUOPL, and research assistants who have completed a medical degree. Through training, all care providers were well experienced in internet-based psychotherapy, with a specific focus on CBT techniques. They were all taught the standard care pathway and the aim, and content of each therapeutic session. They also continued receiving specialized training through webinars, CBT workshops led by a psychiatrist trained in CBT, and exercises with feedback during the study. Moreover, they were provided predesigned feedback templates tailored to each session to be used when writing the weekly feedback. The feedback templates helped standardize the feedback by providing a basic structure that ensured all aspects of the homework were acknowledged and that the newly presented CBT concepts were reviewed. Feedback templates varied between sessions, and care providers personalized each template for each participant’s homework. The use of feedback templates that were drafted by the team psychiatrist trained in online psychotherapy allowed some control over treatment consistency across sessions and participants. Before submission to the participant, feedback was always reviewed by another care provider (MSc candidate) trained in psychotherapy and overlooked by a psychiatrist to ensure the quality of the feedback. The team psychiatrist fulfilled the role to overlook the feedback; manage any crisis events if necessary; and provide support in the stepped care model, specifically the highest-intensity intervention, step 4.

### Intervention

#### i-CBT Program

All participants (56/56, 100%) were provided with the same 13-week i-CBT program designed for MDD through OPTT—a secure, cloud-based, digital mental health platform [38]. This i-CBT program, titled “Electronic Cognitive Behavioural Therapy (e-CBT) For Depression,” is a set of predesigned modules created to address depressive symptoms through various CBT techniques to work on cognitive restructuring [46,49-51]. This specific program has undergone validation through previous studies and has been shown to significantly decrease depressive symptoms [47,49]. The program was intended to imitate in-person, standard CBT for MDD in an asynchronous format. In this study, 1 module was assigned to participants on the same day of each week. A module included 20 to 30 slides of CBT content and took approximately 45 to 50 minutes to view (Multimedia Appendix 2). Each module focused on 1 CBT technique (Table 1). Each module followed a similar outline, beginning with an introduction to the topic for the week, an overview of the technique with examples, and concluding with homework related to the weekly technique [50]. The homework consisted of a few questions based on a stressful event that occurred in the participant’s life in the past week and provided participants with the practice of the new CBT technique. Participants were provided with 4 days to asynchronously review the module and submit their homework to their assigned care provider on the OPTT platform. Participant answers were then reviewed by the assigned care provider over 3 days who provided participants with feedback about their answers along with their next session on the assigned day of the week.

**Table 1:** A brief description of each session for the internet-based cognitive behavioral therapy (i-CBT) program, titled “Electronic Cognitive Behavioural Therapy (e-CBT) For Depression,” on the Online Psychotherapy Tool platform.

Module	Title	Description
1	What is depression?	Introduced i-CBT and discussed common depressive symptoms while setting expectations for the course
2	The 5-Part Model	Described the 5-Part Model: connections and interactions between a situation, thoughts, feelings, physical reactions, and behaviors
3	Sleep hygiene	Focused on how to improve rest through sleep habits and offered a variety of techniques
4	Strategies for stressful situations	Provided a general review of practical strategies that can be applied under difficult circumstances, including breathing techniques and activities
5	Thoughts, feelings, behavior, physical reactions, and environment	Explained the 5-Part Model in detail and how modifications to 1 part can influence the remaining 4 parts
6	The thought record	Featured the first 3 columns of the thought record: a tool for challenging ineffective thoughts The first 3 columns included the situation, feelings, and automatic thoughts associated with the situation
7	Automatic thoughts	Explored the function of automatic thoughts and how they affect emotions This included learning how to recognize automatic thoughts and the “hot thought” Common cognitive errors were also discussed
8	Activity scheduling	Described the activity record: a tool for recording weekly activities and finding connections between activities and associated moods
9	Evidence	Back to the thought record, the fourth and fifth columns were explained, which included looking at evidence for and against the most intense thought
10	Alternative and balanced thinking	Concluded the 5-Part Model with the final 2 columns outlining alternative or balanced thinking and rerating feelings
11	Experiments	Introduced a behavioral activation technique of experiments to promote belief in alternative or balanced thinking
12	Action plans	Promoted working on the identified problems using a structured tool called the action plan
13	Review	Reviewed the 12 modules in the program and summarized the key tools and techniques

The feedback was based on predesigned feedback templates created by the lead psychiatrist and i-CBT expert at QUOPL. The feedback for each session followed a similar structure and was delivered as a letter addressed to the participant. The feedback template mirrored the following structure: (1) addressed the participant and thanked them for their work over the week while acknowledging any adverse events that occurred; (2) commented about their mood and sleep quality in the previous week; (3) summarized the content of the module, highlighting the main concepts; (4) summarized the participant’s answers; (5) empathized with the participant based on their shared experiences and encouraged them to use the learned CBT techniques; and (6) thanked them again for their time and provided the participant with a brief introduction about the next module’s content (Multimedia Appendix 3—sample feedback template for session 1). Overall, the feedback itself focused on the participant’s mood and sleep patterns over the week, progress on their weekly goals, and their understanding of CBT concepts. The care providers personalized the feedback template based on the participant’s responses.

The OPTT platform provided participants and care providers with a modality to communicate asynchronously regarding the availability of the next module and any questions or concerns regarding the OPTT operations or the study design. These questions and concerns were limited to the study design and technical issues, rather than personal or therapy-related concerns. Care providers viewed and replied to these OPTT messages at least once a week. For concerns regarding the OPTT platform and technical issues, participants were redirected to OPTT’s technical support team.

#### i-CBT With Stepped Care

Although all participants (56/56, 100%) had access to the i-CBT program, a designated care provider, and weekly feedback, only participants in the stepped care group received additional interventions as per the proposed stepped care model. The stepped care interventions were provided by their care provider or the team psychiatrist associated with their care as required. The structure of the stepped care model used in this study followed the 4 identified steps of a preventative stepped care model based on 10 randomized controlled trials [52]. These four steps included the following: (1) watchful waiting, (2) self-help psychotherapy, (3) face-to-face psychotherapy, and (4) referral to specialists. The intervention that participants received was dictated by the participant’s care team: the care provider and the team psychiatrist. This model initiated all participants at the lowest intervention of i-CBT beginning at session 1 and provided additional interventions beginning at session 5 after monitoring their progress through the first 4 sessions and obtaining 2 PHQ-9 scores as a quantitative assessment measure, to help with the decision (Textbox 1). Subsequently, an intervention could be added or changed in each session by the care team, following session 5. Beginning stepped care at session 5 allowed for a watchful waiting period as the initial step to understand the patient’s symptoms better, as seen in preventative stepped care models for anxiety and depressive disorders [52]. The first 3 interventions, steps 1 to 3, are similar in content and care provider, but the delivery modality varies. These first 3 steps included varying intensities to care and effort required by the care provider. Step 4 differed from the first 3 steps in that the team psychiatrist was involved and the content of care in this intervention was different. Details of each step in the intervention are provided in the following sections.

#### Step 0: i-CBT Only

This intervention was considered to have the lowest intensity because the participant was provided with an i-CBT program for depression that is effective in treating MDD [49]. This was also known as the starting point for all participants. All participants began at step 0, which included completing the i-CBT program. In this step, no intervention was added to the participants’ care, which allows for patient monitoring during a watchful waiting phase as they receive a low-intensity treatment [52]. Furthermore, many studies have control groups that encompass waitlist groups; however, more studies should focus on implementing i-CBT treatments as the control group for a wholesome review of the effects of stepped care models placed in these i-CBT programs. Therefore, step 0 mimics the procedure for the control group to allow for comparable effects between the 2 groups.

#### Step 1: i-CBT With Messaging

This intervention was the first added intervention following step 0 and included the addition of asynchronous check-in messages to the participant from their care provider on the OPTT platform. When the care provider sent the next session, they also sent a personalized message based on the message templates for that weekly session (Multimedia Appendix 3—sample message template). The message focused on addressing the participant and checking in with them by asking them how their week was so far. This intervention was a brief exchange between the participant and their care provider and a way to add active human support to the participant’s care through direct messaging [53,54]. Upon receiving the participant’s response, care providers acknowledged their experiences and reminded them to complete their next session.

#### Step 2: i-CBT With a Telephone Call

This intervention included a brief telephone call from the care provider for live, verbal support [55-57]. The call is limited to one 15 to 20–minute call during the week [58]. After sending the weekly session, care providers called the participant before the next session. The telephone call focused on asking participants how their week was and acknowledging the participant’s experience (Multimedia Appendix 3—sample call template). Care providers also asked for updates regarding module completion for the week and reminded participants to submit their weekly homework. This template was similar to that of step-1 messages, except that it included direct verbal encouragement.

#### Step 3: i-CBT With a Video Call

This intervention included a brief, secure, Microsoft Teams video call from the care provider for live support and visual contact between the participant and care provider, in addition to verbal cues. The video call is limited to one 20 to 30–minute call during the week [22,59]. Care providers set up a video call with the participant before the next session. The video call focused on the participant’s experience over the past week and how they are doing (Multimedia Appendix 3—sample message template). Care providers also encouraged participants to submit their weekly homework if they had not submitted yet. This template was similar to that of step-2 telephone calls and included direct, live encouragement in hopes to mimic a face-to-face setting.

#### Step 4: i-CBT With Psychiatrist Call

This intervention was the highest-intensity intervention provided in the stepped care model. This intervention included a web-based, one-on-one psychiatrist appointment with the team psychiatrist using Microsoft Teams. The psychiatrist discussed the participant’s current challenges and potential options for the participant. This may include the possibility of adding medication to the participant’s care based on the severity of the case. The selection of medication was based on the Canadian Network for Mood and Anxiety Treatments guidelines, and these guidelines were referenced to decide the first or second line of treatment for each participant [60].

When deciding the appropriate intervention, a participant’s care was not always increased sequentially; instead, a participant who exhibited severe symptoms by the end of session 4 was provided a high step as opposed to step 1. The decision about which intervention the participant would receive was dependent on a few factors considered by the care providers. These included (1) changes in participant’s PHQ-9 scores, (2) engagement with treatment, (3) progress in weekly goals, and (4) homework submission.

### Changes in PHQ-9 Scores

PHQ-9 scores for each participant were collected every 3 weeks (ie, weeks 1, 4, 7, 10, and 13). The initial step-up decision was based on the first 2 PHQ-9 scores collected at weeks 1 and 4. If the participant’s PHQ-9 score at week 4 increased by >2 points compared with week 1, the participant was stepped up in their care to either step 1 (message) or step 2 (phone call). During the subsequent weeks, changes in the subsequent PHQ-9 scores were compared with the previous week’s score (ie, increase of >2 points) to determine whether an additional intervention was required [61].

### Treatment Engagement

A systematic review including 35 studies showed that great treatment engagement significantly improved postintervention mental health outcomes [62]. Thus, participant engagement with treatment through the OPTT platform was another factor that was considered when determining which intervention would be most suitable for the participant. If the participant exhibited limited interaction with the platform (eg, read receipts for messages indicating that messages were not seen when notifying participants about session availability) or expressed difficulties in navigating the platform, the participant was provided with the step-2 (phone call) intervention. This limited engagement indicated that step 1 (messages) would be ineffective because the participant was not viewing the intervention. Therefore, the step-2 (phone call) intervention was identified as a more effective modality than the step-1 (messages) intervention. In addition, if participants did not respond to step-1 (messages) intervention, step-2 (phone call) interventions were provided as a follow-up.

### Homework Submission

Participants’ answers to weekly homework were also considered as a factor when deciding their care intensity [63]. If participants indicated ongoing depressive symptoms that have not changed within the past 2 sessions, they were stepped up in care intensity. Similarly, if depressive symptoms did not improve within the past 2 weeks and if they did not find the current intervention of steps 1, 2, or 3 helpful, step 4 (psychiatrist support) was provided to the participant. Finally, if the care provider sensed any form of suicidal or homicidal ideation or any severe depressive behavior at any point, step 4 (psychiatrist support) was referred to the participant.

### Goal Progress

Beginning in session 1, participants were encouraged to set a goal that they would like to achieve by the end of the program. Each week, participants were prompted to complete a small step toward the goal to help them achieve it. The weekly progress toward this goal rather than absolute achievement was used as another indication of whether a step-up in intervention was required [64]. Studies have shown some support for goal planning in positive treatment outcomes for mental health care through assisting the therapeutic relationship by building rapport and allowing for open communication [65,66]. If participants struggled with the progression in their weekly small step for >2 consecutive sessions, they were stepped up in their intervention.

### Data Analysis

A combination of descriptive statistics, independent *t* tests, and ANOVA were used to determine any difference between the primary outcomes across the 2 groups. All analyses were performed at a 1-tailed significance level of α=.05. To determine any significant difference in the clinical questionnaire scores between the 2 groups, ANOVA was used. A 2×5 repeated-measures ANOVA was conducted for PHQ-9 scores, and a 2×3 repeated-measures ANOVA was performed for Q-LES-Q and QIDS scores. The time points at which meaningful differences appear were examined using the Bonferroni post hoc method. If Mauchly test of sphericity was significant, a Huynh-Feldt correction was applied. A chi-square test was conducted to compare treatment compliance across the 2 groups based on the number of participants completing all 13 sessions of the program. An independent samples *t* test was also performed to compare the number of sessions completed by participants in the 2 groups. In addition, an intention-to-treat (ITT) analysis was completed to assess the clinical outcomes of treatment on participants who withdrew prematurely. No intermediate analyses were performed, and all statistical analyses were completed after the trial. IBM SPSS Statistics (version 28) was used to conduct all analyses [67].

## Results

### Participants

**Figure 2:**
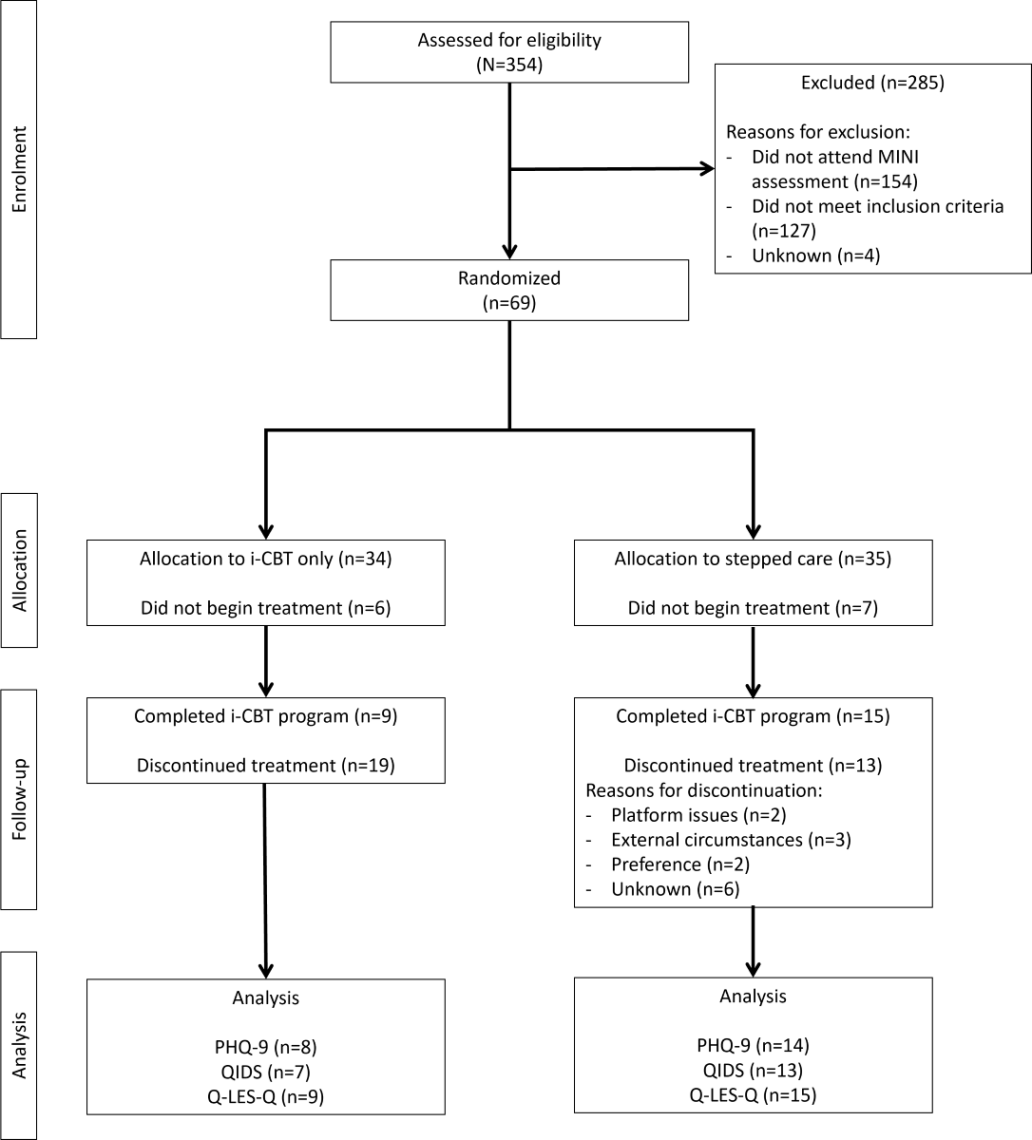
CONSORT (Consolidated Standards of Reporting Trials) flow diagram of participant flow through the study. i-CBT: internet-based cognitive behavioral therapy; MINI: Mini International Neuropsychiatric Interview; PHQ-9: Patient Health Questionnaire–9; QIDS: Quick Inventory of Depressive Symptomatology; Q-LES-Q: Quality of Life Enjoyment and Satisfaction Questionnaire–Short Form.

Recruitment was initiated in May 2021 after receiving approval from the Queen’s University Health Sciences and Affiliated Teaching Hospitals Research Ethics Board. Recruitment was conducted between May 2021 and June 2022, and 69 individuals were found to be eligible for this study. Table 2 displays the participant demographics. Of the 69 eligible individuals, 34 (49%) were randomized to the control group and only 28 (41%) initiated treatment. Of the 28 participants in the control group, 9 (32%) completed the full round of therapy (ie, 13 sessions of i-CBT). Of the 35 individuals randomized to the stepped care group, 28 (80%) initiated treatment. In the stepped care group, only 54% (15/28) of the participants completed all 13 sessions of the i-CBT program. Unfortunately, owing to some data collection errors, some questionnaire scores were not collected during the program, leading to some gaps in pretreatment, midtreatment, or posttreatment scores. These scores were treated as missing and were not imputed during data analysis. Figure 2 shows the participant flow.

Participants in the stepped care group were allocated to the stepped care intervention as follows: step 0 (8/28, 29%), step 1 (message; 2/28, 7%), step 2 (phone call; 15/28, 54%), step 3 (video call; 1/28, 4%), step 4 (psychiatrist consultation; 2/28, 7%). Overall, of the 28 participants, a total of 20 (71%) participants were stepped up in their care. This includes participants who dropped out prematurely and those who completed all 13 sessions. Of the 15 participants who completed all 13 sessions, 12 (80%) participants were stepped up in their care at some point during the program. Table 3 provides information regarding the number of occurrences of each step, and Table 4 provides a summary of each participant’s progression in the stepped care program throughout the treatment, beginning at session 5. Currently, the 1-year follow-up period is ongoing, in which PHQ-9, QIDS, and Q-LES-Q scores are collected at the 3-, 6-, 9-, and 12-month follow-up periods after the treatment. The follow-up period is expected to be completed in November 2023.

**Table 2:** Demographics and characteristics of the participants who began treatment, categorized based on treatment group: internet-based cognitive behavioral therapy (i-CBT) and i-CBT with stepped care.

Characteristics		i-CBT (n=28)	Stepped care (n=28)
Age (y), mean (SD)		37.88 (13.08)	40.57 (14.28)
Baseline PHQ-9^a^ score, mean (SD)		16.63 (4.40)	17.75 (5.33)
**Sex, n (%)**			
	Female	17 (61)	17 (61)
	Male	7 (25)	11 (39)
	Other	2 (7)	0 (0)
	Missing	2 (7)	0 (0)
**Ethnicity, n (%)**			
	Asian	1 (4)	1 (4)
	Hispanic	1 (4)	0 (0)
	White	7 (25)	7 (25)
	Other	17 (61)	20 (71)
	Missing	2 (7)	0 (0)
**First language, n (%)**			
	English	24 (86)	26 (93)
	Hebrew	0 (0)	1 (4)
	Hindi	0 (0)	1 (4)
	Cantonese	1 (4)	0 (0)
	Spanish	1 (4)	0 (0)
	Missing	2 (7)	0 (0)
**Immigration status, n (%)**			
	Born in Canada	12 (43)	13 (46)
	Immigrated to Canada	3 (11)	2 (7)
	Missing	13 (46)	13 (46)
**Employment, n (%)**			
	Full time	13 (46)	15 (54)
	Part time	3 (11)	4 (14)
	Unemployed	6 (21)	8 (29)
	Student	4 (14)	1 (4)
	Missing	2 (7)	0 (0)
**Marital status, n (%)**			
	Married	7 (25)	10 (36)
	Never married	14 (50)	13 (36)
	Divorced	1 (4)	2 (46)
	Widowed	2 (7)	1 (7)
	Other	2 (7)	2 (4)
	Missing	2 (7)	0 (0)
**Children, n (%)**			
	Yes	5 (18)	13 (46)
	No	21 (75)	15 (54)
	Missing	2 (7)	0 (0)
**Income (CAD $; CAD $1=US 0.74), n (%)**			
	<20,000	7 (25)	6 (21)
	20,000-34,999	5 (18)	3 (11)
	35,000-49,999	5 (18)	8 (29)
	50,000-74,999	4 (14)	6 (21)
	75,000-99,999	0 (0)	2 (7)
	>100,000	3 (11)	3 (11)
	Missing	4 (14)	0 (0)

^a^PHQ-9: Patient Health Questionnaire–9.

**Table 3:** Sample size of the highest intervention provided for participants in the stepped care group, sorted based on completion and dropout from the internet-based cognitive behavioral therapy (i-CBT) program.

Stepped care interventions	Step 0: i-CBT only, n (%)	Step 1: message, n (%)	Step 2: phone call, n (%)	Step 3: video call, n (%)	Step 4: psychiatrist, n (%)
Dropout (n=13)	5 (38)	1 (8)	6 (46)	0 (0)	1 (8)
Completed (n=15)	3 (20)	1 (7)	9 (60)	1 (7)	1 (7)
Total (n=28)	8 (29)	2 (7)	15 (54)	1 (4)	2 (7)

**Table 4:** The number of participants provided with each stepped care intervention for each session, from session 5 to session 13 (n=28).

Step	Session 5, n (%)	Session 6, n (%)	Session 7, n (%)	Session 8, n (%)	Session 9, n (%)	Session 10, n (%)	Session 11, n (%)	Session 12, n (%)	Session 13, n (%)
N/A^a^	3 (11)	5 (18)	6 (21)	6 (21)	8 (29)	9 (32)	11 (39)	13 (46)	13 (46)
0	16 (57)	17 (61)	16 (57)	15 (54)	12 (43)	15 (54)	10 (36)	12 (43)	8 (29)
1	4 (14)	1 (4)	2 (7)	1 (4)	4 (14)	0 (0)	3 (11)	1 (4)	2 (7)
2	5 (18)	5 (18)	4 (14)	6 (21)	4 (14)	2 (7)	2 (7)	1 (4)	3 (11)
3	0 (0)	0 (0)	0 (0)	0 (0)	0 (0)	2 (7)	1 (4)	1 (4)	1 (4)
4	0 (0)	0 (0)	0 (0)	0 (0)	0 (0)	0 (0)	1 (4)	0 (0)	0 (0)

^a^N/A: not applicable.

### Measured Outcomes

#### PHQ-9 Score

**Figure 3:**
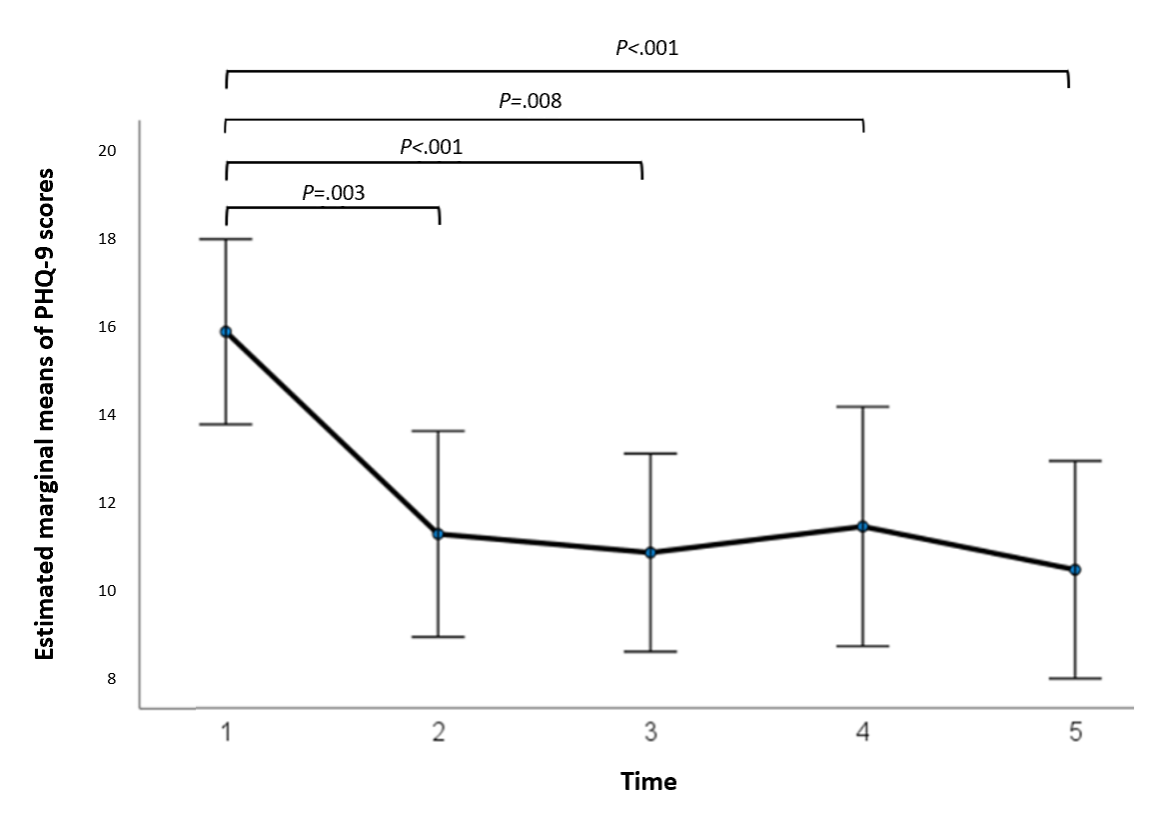
Estimated marginal means of Patient Health Questionnaire–9 (PHQ-9) scores at 5 treatment time intervals corresponding to sessions 1, 4, 7, 10, and 13, including both groups—internet-based cognitive behavioral therapy (i-CBT) only and i-CBT with stepped care. Error bars depict –2 or +2 SE.

**Figure 4:**
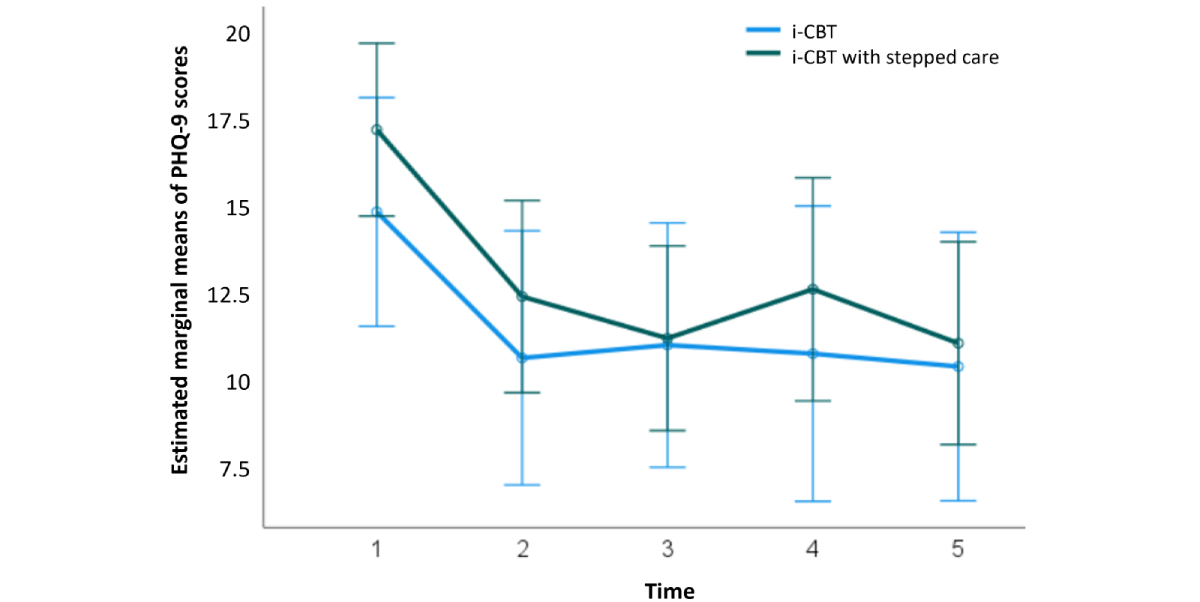
Estimated marginal means of Patient Health Questionnaire–9 (PHQ-9) scores at 5 intervals of treatment (sessions 0, 4, 7, 10, and 13), in both internet-based cognitive behavioral therapy (i-CBT; blue) and i-CBT with stepped care (green) treatment conditions. Error bars depict –2 or +2 SE.

At baseline, for the participants who initiated the i-CBT program, the mean PHQ-9 score was 16.63 (SD 4.40; 27/28, 96%) for the control group and 17.75 (SD 5.33; 28/28, 100%) for the stepped care group, showing no statistically significant difference in pretreatment scores between the 2 groups (*t*_53_=−0.85; *P*=.40; 95% CI −3.77 to –1.53). A 2×5 repeated-measures ANOVA determined that the mean PHQ-9 scores differed significantly between time points (*F*_4,80_=9.95; *P*<.001; Figure 3), but there was no significant difference at different time points between the 2 groups (*F*_4,80_=0.43; *P*=.78; Figure 4). Post hoc analysis with Bonferroni adjustment revealed that PHQ-9 scores significantly decreased from pretreatment period (week 1) to the second time point (week 4; 4.554, 95% CI 1.264-7.843; *P*=.003), from pretreatment period (week 1) to the third time point (week 7; 4.973, 95% CI 1.904-8.042; *P*<.001), from pretreatment period (week 1) to the fourth time point (week 10; 4.384, 95% CI 0.862-7.906; *P*=.008), and from pretreatment period (week 1) to posttreatment period (week 13; 5.357, 95% CI 2.169-8.546; *P*<.001). All between–time point scores were statistically significant (Figure 3). A detailed breakdown of the results are summarized in Multimedia Appendix 4.

#### QIDS Score

**Figure 5:**
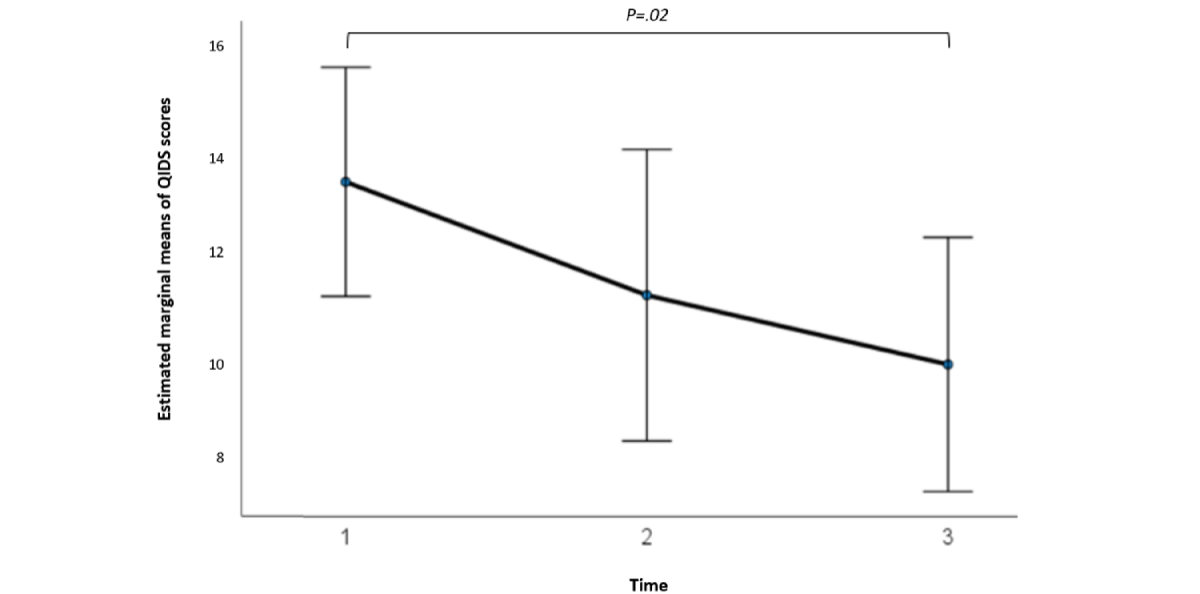
Estimated marginal means of Quick Inventory of Depressive Symptomatology (QIDS) scores at 3 treatment time intervals corresponding to sessions 1, 7, and 13, including both groups—internet-based cognitive behavioral therapy (i-CBT) only and i-CBT with stepped care. Error bars depict –2 or +2 SE.

**Figure 6:**
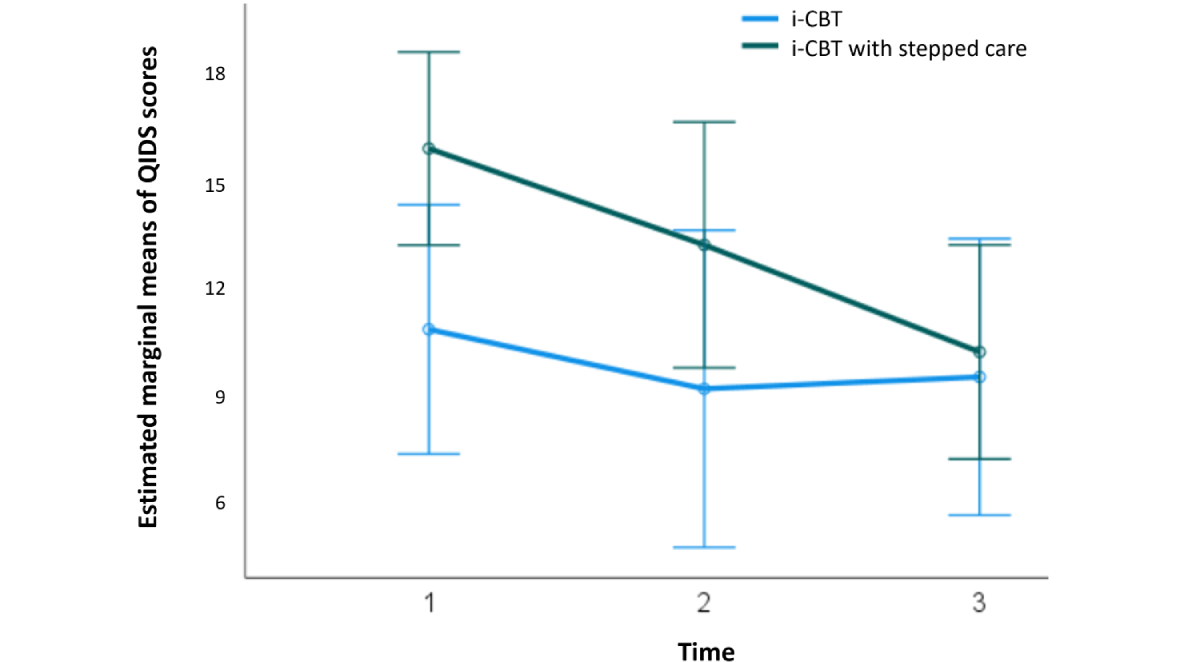
Estimated marginal means of Quick Inventory of Depressive Symptomatology (QIDS) scores at 3 intervals of treatment (sessions 0, 7, and 13), in both internet-based cognitive behavioral therapy (i-CBT; blue) and i-CBT with stepped care (green) treatment conditions. Error bars depict –2 or +2 SE.

At baseline, for the participants who initiated the i-CBT program, the mean QIDS score was 14.62 (SD 4.64; 21/28, 75%) for the control group and 16.83 (SD 4.43; 23/28, 82%) for the stepped care group, showing no statistical significance in pretreatment scores between the 2 groups (*t*_42_=−1.61; *P*=.11; 95% CI −4.97 to 0.55; independent samples *t* test). A 2×3 repeated-measures ANOVA determined that the mean QIDS scores differed significantly between time points (*F*_2,28_=5.73; *P*=.008; Figure 5), but there was no significant difference at different time points between the 2 groups (*F*_2,28_=3.05; *P*=.06; Figure 6). Post hoc analysis with Bonferroni adjustment revealed that QIDS scores significantly decreased from pretreatment period (week 1) to posttreatment period (week 13; 4.12, 95% CI 0.76-7.47; *P*=.02). There was no significant difference between pretreatment period (week 1) and midtreatment period (week 7; 2.08, 95% CI −1.10 to 5.26; *P*=.29) or between midtreatment period (week 7) and posttreatment period (week 13; 2.03, 95% CI −1.34 to 5.41; *P*=.37; Figure 5). A detailed breakdown of the results are summarized in Multimedia Appendix 4.

#### Q-LES-Q Score

**Figure 7:**
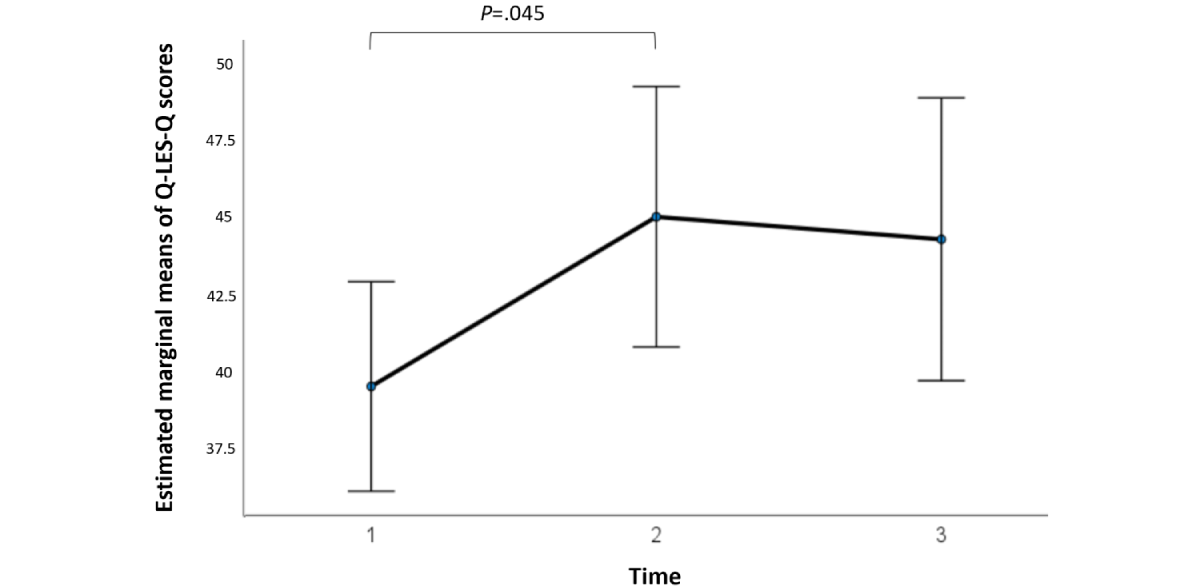
Estimated marginal means of Quality of Life Enjoyment and Satisfaction Questionnaire–Short Form (Q-LES-Q) scores at 3 treatment time intervals corresponding to sessions 1, 7, and 13, including both groups—internet-based cognitive behavioral therapy (i-CBT) only and i-CBT with stepped care. Error bars depict –2 or +2 SE.

**Figure 8:**
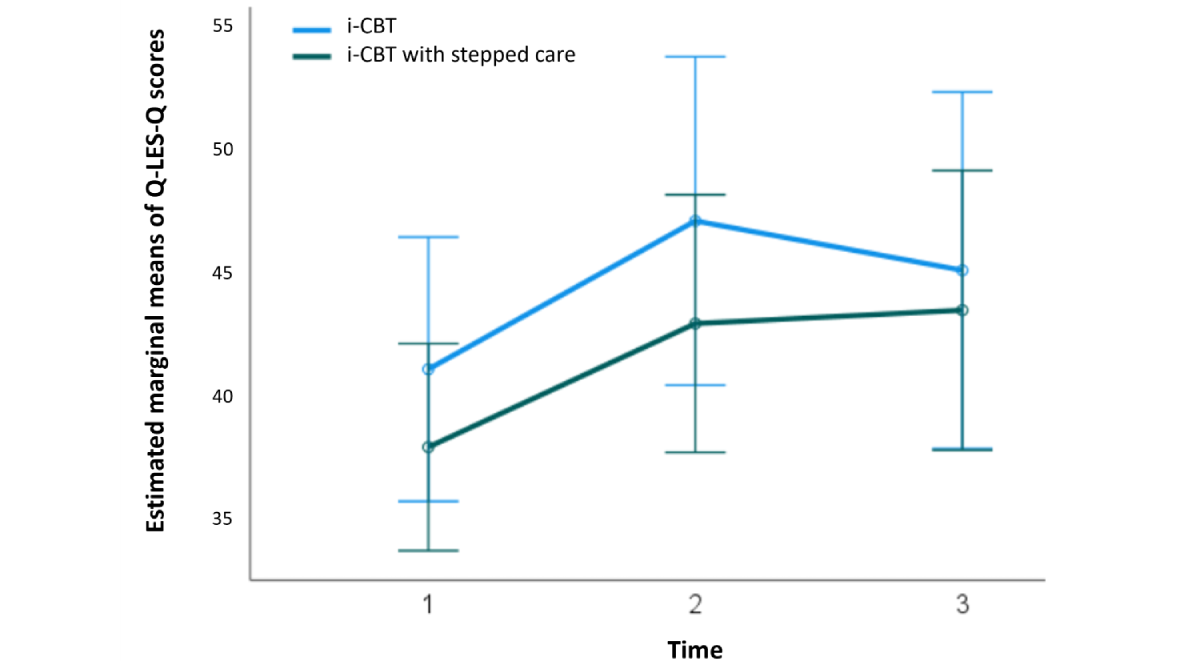
Estimated marginal means of Quality of Life Enjoyment and Satisfaction Questionnaire–Short Form (Q-LES-Q) scores at 3 intervals of treatment (sessions 0, 7, and 13), in both internet-based cognitive behavioral therapy (i-CBT; blue) and i-CBT with stepped care (green) treatment conditions. Error bars depict –2 or +2 SE.

At baseline, for the participants who initiated the i-CBT program, the mean Q-LES-Q score was 36.88 (SD 7.80; 26/28, 93%) for the control group and 36.25 (SD 7.81; 28/28, 100%) for the stepped care group, showing no statistical significance in pretreatment scores between the 2 groups (*t*_52_=0.30; *P*=.77; 95% CI −3.63 to 4.90; independent samples *t* test). A 2×3 repeated-measures ANOVA determined that the mean Q-LES-Q scores differed significantly between time points (*F*_2,38_=4.18; *P*=.02; Figure 7), but there was no significant difference at different time points between the 2 groups (*F*_2,38_=0.19; *P*=.83; Figure 8). Post hoc analysis with Bonferroni adjustment revealed that Q-LES-Q scores significantly increased from pretreatment period (week 1) to midtreatment period (week 7; −5.50, 95% CI −10.91 to –0.94; *P*=.045). There were no significant differences between pretreatment period (week 1) and posttreatment period (week 13; −4.77, 95% CI −11.03 to 1.49; *P*=.18) and between midtreatment period (week 7) and posttreatment period (week 13; 0.73, 95% CI −5.18 to 3.72; *P*=.99; Figure 7). A detailed breakdown of the results are summarized in Multimedia Appendix 4.

### Treatment Compliance

The proportion of participants completing the full round of therapy (ie, 13 sessions) was 32% (9/28) in the i-CBT–only group and 54% (15/28) in the stepped care group. There was no significant difference between the 2 groups regarding completion of the i-CBT program for depression (*χ*^2^_1_=2.6; *P*=.10; chi-square test). An independent samples *t* test was conducted to determine whether there was a difference in the number of completed sessions between the i-CBT group and the stepped care group for the individuals who had begun the program. The results indicate significant difference between the average number of sessions completed by participants in the i-CBT–only group (mean 7, SD 4.64; 28/28, 100%) and stepped care group (mean 9.41, SD 4.44; 28/28, 100%; *t*_55_=−2; *P*=.03; 95% CI −4.83 to −0.002; independent samples *t* test). Multimedia Appendix 5 shows the distribution of the sessions completed in each group.

### ITT Analysis

A linear mixed model analysis was used to conduct the intent-to-treat analysis for each participant across the program. The group (i-CBT or stepped care) and evaluation time points were indicated as fixed factors. This analysis included participants who did not complete the full program and dropped out of the study before completion. As seen previously, there was significant change in PHQ-9 scores across the time points (*F*_4,48.79_=10.98; *P*<.001), but there was no significant difference between the 2 groups (*F*_1,107.07_=2.79; *P*=.10). In addition, for the QIDS scores, there was significant change in scores across the time points (*F*_2,41.08_=8.53; *P*<.001), but no significant difference between the 2 groups (*F*_1,54.44_=2.72; *P*=.10). Q-LES-Q scores imitated a similar pattern, with significant difference in scores across the time points (*F*_2,44.99_=9.36; *P*<.001), but no significant difference between the 2 groups (*F*_1,60.75_=3.28; *P*=.08).

## Discussion

### Principal Findings

This study evaluated the effectiveness of an i-CBT program with and without stepped care for depression among adults. The results of this study indicate that the proposed stepped care model was not significantly better in reducing depressive symptoms, as measured by PHQ-9 and QIDS, than the i-CBT program alone. Some previous studies have also found no significant differences between stepped care treatments and care as usual in improving depressive symptoms [52,68]. Ho et al [52], who reviewed stepped care for both depressive and anxiety disorders, found that stepped care treatment was significantly better in improving anxiety symptoms but did not find any significant difference in reducing depressive symptoms. This suggests some differences in the populations and the sensitivity of the stepped care structure in addressing different mental health issues. When viewing the results of both groups together, this study indicated that the i-CBT program itself was effective in significantly reducing depressive symptoms from pretreatment period to posttreatment period, as measured by the PHQ-9 and QIDS; however, there were no significant differences in the reduction of depressive symptoms between the 2 groups. The stepped care group did not show to be significantly better in reducing depressive symptoms than the i-CBT group. This finding is consistent with other studies of i-CBT programs, which show improvements in depressive symptoms for mild to moderate depression [69,70]. This also reinforces the efficacy of the i-CBT program for depression on OPTT used in this study, based on similar results from previous clinical trials using this program [47,49].

Furthermore, the results suggest that the i-CBT program indicated significant difference in the quality of life, as measured by Q-LES-Q; however, post hoc analysis revealed that overall, there were no significant differences before and after treatment in improving the quality of life. Significant difference was observed between pretreatment period and midtreatment period, which may suggest that the i-CBT program had the greatest impact on improving quality of life in the early stages of the program. No significant differences were observed between the i-CBT and stepped care groups. A systematic review and meta-analysis reviewed 3 studies of the effects of i-CBT on the quality of life and found inconclusive evidence of i-CBT compared with in-person outcomes [71]. We found no significant difference between the effects of i-CBT with stepped care and i-CBT without stepped care on the quality of life.

Although the completion rates between the 2 groups were not significantly different, participants in the stepped care group significantly completed, on average, 2 more sessions of therapy than those in the i-CBT group. The increase in the number of completed sessions may have been caused by the added support offered in the stepped care group. This is consistent with previous studies that report improvement in treatment adherence in mental health treatment with additional interventions in care [21,28,63]. Specifically, phone and video calls assist with keeping patients in care compared with in-person treatment [36]. Compared with in-person treatment, receiving care for depression over the phone [56,72] or via video [22] is associated with high completion rates and few dropouts. Further studies are required to decipher whether the stepped care group’s high completion rate of sessions is associated with better treatment outcomes than the i-CBT group.

Furthermore, Multimedia Appendix 5 shows that although participant dropout occurs uniformly across the first 10 sessions in the stepped care group, most participants (19/28, 68%) in the i-CBT group dropped out in the first half of the program (ie, first 7 sessions): 25% (7/28) of participants in the stepped care group dropped out of the program before the midpoint of treatment (ie, session 7) and 68% (19/28) of participants in the i-CBT group. Previous studies have noted that most patients drop out of treatment programs after 2 to 4 sessions [16,31,73]. During this time, participants were provided with i-CBT care only in both groups (additional interventions for the stepped care group were introduced at session 5 after monitoring their PHQ-9 scores and interaction with the i-CBT program); however, introducing a possibility of intervention soon in the stepped care group may have helped participants who were indicated as an early dropout (ie, before session 5) complete more sessions. Concurrently, it is noteworthy that the i-CBT program’s 13 sessions were not completed by several participants (32/56, 57%) in both groups. This is consistent with previous studies that reveal high dropout rates in depression i-CBT programs. Compliance is generally a challenge for i-CBT programs, with dropout rates averaging approximately 32% and ranging between 0% and 75% [26,37], whereas traditional CBT dropout rates are approximately 25% on average, ranging between 0% and 68% [74]. However, another study found that additional human support results in a large effect size regarding the efficacy of i-CBT (g=0.673) than providing no additional support (g=0.239) [34]. Thus, it was expected that the stepped care group would exhibit significant improvements following treatment, but this was not observed in this study. This may be owing to the nature of the study design, as the i-CBT group received some support during the treatment through the homework feedback and did not encompass a truly no-support intervention. Upon reviewing participant progression in the stepped care model (Table 4), it was seen that on average, most participants (13/28, 46%) spent their time in step 0 of the stepped care model, which mimics the control group (i-CBT only). This may be a factor in the observed nonsignificant differences between the 2 groups because not many participants were stepped up in their care across treatment. Moreover, the small sample size may have influenced the outcomes. A previous study has shown that patients who terminate CBT prematurely show high symptom severity compared with patients who complete therapy, but the 2 groups did not vary in the rate of symptom change [75]. This suggests that the increase in symptom severity may arise from completing few sessions of treatment in the dropout group compared with the completer group and explains the differing results observed between the 2 groups as dropout was high. Given the effectiveness of i-CBT treatment and the high dropout rates, it is important to assist patients in completing more sessions by providing sufficient resources and care. Our proposed stepped care model assisted participants in completing a great number of sessions. Stepped care has been associated with high treatment satisfaction, which may have assisted in participants completing more sessions in this group [68,76]. Future studies should examine the variables that may be responsible for treatment attrition in i-CBT programs and devise methods to raise the rates of treatment engagement and completion.

### Limitations

It is important to consider further limitations of this study. This includes the relatively small sample size; observed sex imbalance, with 61% (34/56) women in the study (approximately 15% more women in the i-CBT group compared with the stepped care group); and lack of long-term follow-up, which is currently ongoing. It is important to note that the sample size for analysis of the i-CBT group was smaller than that of the stepped care group. In addition, the participants in this study may not be representative of the general population, as they were predominantly women, English speaking (owing to the limitations of the i-CBT program), employed full time, and recruited from a specific clinical setting of self-referrals and clinics limited to Kingston, Ontario (Table 2). Missing data were also a challenge that affected the total sample size. A large portion of the data was unavailable owing to collection errors and dropouts (Figure 2). We attempted to address this issue by using ITT analysis and observed similar results with no significant differences between groups but observed significant differences across various time points for PHQ-9, QIDS, and Q-LES-Q scores.

This study was not specifically designed to investigate the effects of the different treatment interventions in the stepped care group and how they influenced the results. In hindsight, using 4 different factors to decide about the stepped intervention results in increased variability in the stepped care decision and limits our ability to decipher the effectiveness of the approach. Most stepped care models use clinical questionnaire scores, such as the PHQ-9 score, to decide when to step up or step down care [68,76-78]; however, our study did not include strict cutoff guidelines and instead adopted a subjective monitoring approach of PHQ-9 scores as one of the evaluating factors. Changes in PHQ-9 scores (scale ranging from 0-27) and homework submission (submitted or not submitted) can be quantized predictors for stepped care; however, monitoring engagement with the OPTT platform and goal progression are variables that are multifactorial and are subject to interpretation. This design prevented us from making conclusions about a best-fit intervention model. Furthermore, beginning the stepped care interventions in session 5 allowed for a watchful waiting period that allowed reflection about patient status to determine the correct intensity of care if they did not improve with a low-resource intervention (ie, i-CBT). However, this may be a limitation of the proposed stepped care model, as the waiting period may be harmful because it may delay optimal treatment [68]. With a small sample size, it is difficult to analyze the effects of these deciding factors. Future studies need to be conducted to explore the effects of the individual stepped interventions provided in this study and any potential relationships among the modalities of care.

Therapeutic alliance in psychotherapy is another significant factor in predicting treatment outcomes [79-81]. The i-CBT–only group had limited interaction with their assigned care provider, and the ability to build rapport with participants was limited. In the stepped care group, participants were potentially able to gain a deep connection with their care provider, beginning in session 5, based on the intervention provided to them. Therapeutic alliance follows two important phases: (1) initial alliance development, usually occurring within the first 5 sessions, and (2) challenging the patient more actively, which can cause strain to the therapeutic relationship [79]. With stepped care interventions being provided in session 5, the first phase seems to be neglected and may affect the potential relationship, thus the structure of the program hinders participants from achieving the desired treatment outcome and reducing their depressive symptoms. Some studies demonstrate that a positive outcome is more predictive by the quality of the alliance rather than the type of the intervention [79,82-84]. In this case, the quality of the alliance was affected by the limited interaction with the care provider and may reflect one of the limitations of online therapy. Some studies found that building therapeutic alliances face to face is significantly more effective than online psychological treatments [85-89]. In contrast, other studies have found the opposite and report no correlation between the therapeutic alliance and treatment outcomes in digital interventions [90-93]. Further studies are required to better understand the characteristics of therapeutic alliance in digital contexts [25,94]. It would also be interesting to assess participant and care provider opinions about their therapeutic alliance during the study to better understand this limitation.

### Conclusions

This study provides further evidence that i-CBT is effective for treating depressive symptoms. However, the study showed no significant evidence that the proposed stepped care model was more effective than i-CBT alone. The stepped care model allowed participants to complete 2 more sessions on average than the i-CBT–only group, indicating that stepped care is an effective method for guiding patients to treatment completion. Future studies should examine the long-term effects of such interventions and the efficacy of specific stepped care interventions in large and more diverse groups. To improve the design and implementation of such a model, studies might also investigate the processes through which stepped care interventions reduce depression symptoms and enhance the quality of life.

## Appendix

Multimedia Appendix 1 CONSORT-eHEALTH checklist (V 1.6.1).

Multimedia Appendix 2 Sample of the e–cognitive behavioral therapy program for depression used in the study, showcasing session 5.

Multimedia Appendix 3 Feedback templates for e–cognitive behavioral therapy used throughout the study by care providers including feedback for session homework and live interactions between the care provider and participants.

Multimedia Appendix 4 Statistical analysis results.

Multimedia Appendix 5 The frequency of participants’ last completed sessions organized by 2 groups—internet-based cognitive behavioral therapy (i-CBT) only and i-CBT with stepped care.

## Abbreviations

CBT: cognitive behavioral therapy
CONSORT-EHEALTH: Consolidated Standards of Reporting Trials of Electronic and Mobile Health Applications and Online Telehealth
i-CBT: internet-based cognitive behavioral therapy
ITT: intention-to-treat
MDD: major depressive disorder
MINI: Mini International Neuropsychiatric Interview
OPTT: Online Psychotherapy Tool
PHQ-9: Patient Health Questionnaire–9
Q-LES-Q: Quality of Life Enjoyment and Satisfaction Questionnaire–Short Form
QIDS: Quick Inventory of Depressive Symptomatology
QUOPL: Queen’s Online Psychotherapy Lab

## Data Availability

The data sets generated during and analyzed during this study are available from the corresponding author on reasonable request.
