# Supplementary material for: Incorporating a Stepped Care Approach Into Internet-Based Cognitive Behavioral Therapy for Depression: Randomized Controlled Trial
Source: JMIR Ment Health. 2024 Feb 9;11:e51704. doi: 10.2196/51704 (PMC10891491; doi:10.2196/51704)
Supplement: Multimedia Appendix 3 [file mental_v11i1e51704_app3.docx]

### Sample Feedback Template for Session 1.

# Session 1: What is Depression?

*Assignment Questions:*

1. Tell me your story. What are your concerns at this time? What are your challenges? What are you hoping for?
2. Choose a SMART goal.
3. What is your small step?

Hello _________, *(participant’s name)*

My name is ____________, (your full name) and I am your care provider for this program. I work under Dr. Megan Yang’s supervision and Dr. Nazanin Alavi's leadership. Dr. Alavi is a psychiatrist at Queen's University and the lead of Queen’s University Online Psychotherapy Lab (QUOPL) https://www.queensopl.ca/team-quopl. Dr. Megan Yang is a psychiatrist at Queen’s University and a member of QUOPL. We will be your care team throughout this course. Thank you for taking the time to send in your assignment and for sharing your story with me. I truly appreciate the time and effort you put in to sharing your experiences. I understand it is not easy and that you have been dealing with a lot recently.

Depression can make you feel very sad, and you may not enjoy your hobbies as much as before, your energy might be low, and you might notice changes in your sleep and appetite. These feelings might not go away for a long time. You might feel like you are all alone, worry about death, cry, or feel angry. Sadness can make your body feel different. This could include headaches, stomach aches, feeling sweaty or even nauseous or throwing up or having a pounding heart. Your body might feel really heavy and slow, and you might feel like you cannot make yourself do anything. If you take a step back and look at all the stressors in your life, you can see how they can cause you to feel depressed and stressed out. These stressors can cause you to experience different thoughts and some of them might be negative and unrealistic. We experience distorted thoughts when we have anxiety and/or depression. These thoughts will make you more depressed and as a result experience what you explained. Sometimes it might not be easy to connect the dots but if you look closely all these symptoms may be connected.

You discussed some challenges that you are currently facing and things you would like to improve on, such as *(include parts of ‘Share your story.’, summarize their challenges and depressive symptoms here)*... Overall, you want to *(briefly summarize their goal(s) for themselves that they have listed, can use ‘Specific’ part of SMART goal here)...* We will work on these aspects throughout the course and help you get back to managing your day and feeling more like yourself!

There are tools in this course that we will go through that will help you break down and understand your thoughts, feelings, physical reactions, and behaviours, as well as learn how to change these negative thoughts into more effective ways of thinking. Together we can go through the stressful situations and overwhelming parts of your life and work through them so that you can handle stress better and feel more motivated. It’s also good to think of a friend or family member that you find you can talk to about how you’re feeling. Learning to communicate your feelings and just having someone that you feel comfortable sharing your feelings and thoughts with can help ease some stress. We will work on these aspects throughout this course and help you get back to managing your day and feeling better.

In this session we discussed setting goals for therapy as an important factor in changing behaviour. Specifically, when determining a goal, we follow the SMART goal criteria. **SMART** is an acronym that helps us set up goals that are achievable and realistic. SMART goals are **Specific**: the goal is well defined and clear to anyone; **Measurable**: the goal allows for accurate tracking of progress so you know when you have met your goal; **Achievable**: the goal should be manageable for you; **Relevant and Realistic**: the goal should be applicable to the issue you would like to address and benefit you directly; and **Time-Based**: the goal should have a set timeframe to further quantify your goal and keep you on track.

Your **SMART** goal was centered around *(include ‘Specific’ part of SMART goal*... To develop a SMART goal, it is helpful to be specific about what you are trying to work on. With a generic goal it can be hard to identify how you will know that you are completing it. *(provide feedback on the goal and ‘specific’ aspect as needed here; ex., if not specific enough, indicate and provide suggestions, etc).* You’ve chosen a great goal to set for yourself and this is something many people struggle with, but we will work on this together. This goal is measurable since you will *(include ‘Measurable’ part of SMART goal)*... You indicated this goal is achievable *(adapt this ‘Achievable’ part of SMART goal if needed)* and you have already started working on this goal by taking this course and seeking help. Good for you! It is realistic and relevant since it is something that will benefit you directly and you will *(adapt this ‘Realistic/Relevant’ part of SMART goal if needed)*... Lastly, setting a timeframe for your goal is a good practice so that you know if you are on track. You mentioned a timeframe of *(adapt this ‘Time-based’ part of SMART goal as needed)*... I’m hoping the 13 sessions of this course will help you achieve this goal, and we will work on this together! Your small step for this week was to *(include the small step part here)* … Overall, well done on providing a SMART goal and coming up with a small step!

I’m hoping the 13 sessions of this course will help you with your journey of achieving your goal, and we will work on this together! CBT is a very structured kind of therapy; it is the gold standard and the most effective evidence-based therapy for treatment of anxiety and depression. You will gradually see a connection between your thoughts, symptoms, and behaviours, and learn to change the way you think and behave in stressful situations, which will gradually improve your mood. CBT works specifically on challenging your thoughts and beliefs to a more effective way of thinking. As long as you are open and willing to learn the skills this course has to offer, we can slowly address your concerns and find tools that will help you in stressful situations. The purpose of the weekly questions at the end of the sessions is to help you apply and practice the techniques we learn in the session. If at any point you find that this program is not working for you and you would like to stop, or if you have thoughts on how we can support your progress, please don't hesitate to let me know. Like any therapy, collaboration can make a big difference. It can take some work to challenge some of your negative thoughts, but I am here to help you through this process, and I am hopeful that we will be able to work towards shifting your negative self-talk to something more effective.

I want to remind you that this program is designed to help you learn new techniques and strategies in stressful situations and is not designed for a crisis. If you experience suicidal ideation or feel that you need more help or support please call 911, or the 24-hour crisis line 613-544-4229, as we are not available all the time and we check the platform and messages once a week. We want to make sure that you are safe and get the best help you need and deserve.

Together I’m hoping we can help you feel better and find the tools that work best for you! 😊 As long as you are open and willing to learn the skills this course has to offer, we can slowly address your concerns and find tools that help in stressful situations. I’m looking forward to working with you, ___________. Take care in the meantime.

Regards,

______________ (your name)

###

### Sample Message Template

*Hi ______. I hope you are having a great week. I just wanted to check in with you and see how you are doing. How has your week been so far?*

*…*

*Thank you for sharing that with me. [Acknowledge what the participant has shared]*

*I wanted to remind you to submit this week’s session by __________ [indicate day]. I am looking forward to reading through your homework and working on it together. Have a great day _____!*

###

### Sample Call Template

**** When you phone call the participant, make sure you enter **67* before you dial the number; this hides your phone number and name.**

**Initial Contact – Call in the beginning of the program to set up a recurring day/time.**

*Hi, am I speaking to (participant name) …* [Make sure you are talking to the right person before continuing.]

*My name is ___________ and I am your care provider for the OPTT AI depression study. I will be working with you for the duration of the 13-week program. How are you doing today?*

*I work under Dr. Nazanin Alavi’s supervision, she is the primary investigator for this study, and she is a psychiatrist at Queen’s University. I have sent out the first session and a few questionnaires on OPTT for you to complete. If you have any questions or concerns, please don’t hesitate to bring them up at any point. You can discuss them in our weekly calls or send them through the OPTT messaging platform and I will take a look at the messages every* indicate the day you will look at the messaging platform (I.e., Monday)*.*

*I will be checking in with you every week through either a phone call or a video call. Which one would you prefer? … Ok great, and I would like to schedule a day of the week and time that works best for you. Is there a certain day and time that works best for a weekly 15–20-minute call? … Great. I will* call/video call *you on __________* (I.e., Mondays at 1 pm)*.*

**[For phone calls:]** *I will call from a private number so make sure to pick it up.*

**[For video calls:]** *I will send you a Teams video meeting link for the video call.*

*If anything changes, you can contact me through the OPTT messaging platform to let me know. I look forward to working with you throughout this program. Do you have any questions?*

*Great, have a great day and take care ___________. Bye!*

**Weekly Telephone/Video Call:** *check-in, remind strategies + remind to complete session/due date (encouraging template)*

**[15-20 minutes total]**

***(5-10 mins):***

*Hi, am I speaking to __________? …* [Make sure you are talking to the right person before continuing – this is more for the first few sessions as you start to recognize the voice but always ensure you are addressing the right person; in video call you can see them so adapt this to be more natural].

*Hi, it's* (your name) *from OPTT for the AI depression study.* [in video call you can see them so adapt this to be more natural] *In today’s phone call I want to take a few minutes to check in with you. How are you doing today and how has your week been so far? …* [Listen and acknowledge the difficulties they had in the past week]. *That sounds like a* difficult/nice/relaxing [choose a good reflection word to summarize what they shared] *week.*

*When we go through a difficult time and feel depressed, it’s easy to dismiss good moments. I was wondering if you can try to think of 1 good thing that may have happened in the past week?* … [Listen and acknowledge their experience].

***(5-10 mins):***

*Thank you for sharing _______! I also want to spend some time today to go over any questions you have regarding the session’s content and techniques we have learned so far. Do you have any questions about this week’s session? … Are there any questions about CBT that you have in general or regarding the previous sessions? … How are you finding the strategies you have learned so far? …* [Answer any questions they have]

*Ok great, I want to finish off with a quick review of this next session and what to expect. [Summarize next week’s session, i.e., what it is about and the main concept that will be taught - 5 Part Model, Sleep Hygiene, Strategies for stressful situations, Thought Record, Experiment, Action Plan] Do you have any questions? …* [Answer any questions they have]

*Great, as a reminder your next session is due on* (include due date for session - I.e., Monday, November 21 at 11pm)*. I will call you again next week on* [indicate date and time - I.e., Monday at 1pm] *- does that work for you? …*

*It was great talking to you today _________ and thank you for your time. I hope you have a great day and take care.*

*Bye.*

**Don’t forget to make note of how long the phone call was, and the date you called them.*

## 
