# Supplementary material for: Incorporating a Stepped Care Approach Into Internet-Based Cognitive Behavioral Therapy for Depression: Randomized Controlled Trial
Source: JMIR Ment Health. 2024 Feb 9;11:e51704. doi: 10.2196/51704 (PMC10891491; doi:10.2196/51704)
Supplement: Multimedia Appendix 4 [file mental_v11i1e51704_app4.docx]

**MULTIMEDIA APPENDIX 4. Statistical Analysis Results**

|  | | | | | | | | **Within Subjects Effects** | | | | | |
| --- | --- | --- | --- | --- | --- | --- | --- | --- | --- | --- | --- | --- | --- |
| **Variable** | **Condition** | ***N*** | **Session 0**  **(Baseline)** | **Session 4** | **Session 7**  **(Mid-Treatment)** | **Session 10** | **Session 13**  **(Post-Treatment)** | ***Time*** | | | ***Time by CBT Group Interaction*** | | |
|  |  |  | ***M, SD*** | ***M, SD*** | ***M, SD*** | ***M, SD*** | ***M, SD*** | ***df*** | ***F, p*** | ***ηp^2^*** | ***df*** | ***F, p*** | ***ηp^2^*** |
| PHQ-9 | i-CBT | 8 | 14.75, 4.10 | 10.50, 4.10 | 10.88, 4.19 | 10.62, 5.37 | 10.25, 5.18 | 4, 80 | 9.95,  <.001 | .332 | 4, 80 | .433, .785 | .021 |
|  | sc-CBT | 14 | 17.14, 5.01 | 17.14, 5.01 | 11.07, 5.43 | 12.50, 6.43 | 10.93, 5.70 |  |  |  |  |  |  |
| QIDS | i-CBT | 6 | 10.83, 2.79 | N/A | 9.17, 4.62 | N/A | 9.50, 3.89 | 2, 28 | 5.73,  .008 | .290 | 2, 28 | 3.047,  .063 | .179 |
|  | sc-CBT | 10 | 17.10, 4.77 |  | 14.60, 5.60 |  | 10.20, 5.16 |  |  |  |  |  |  |
| Q-LES-Q | i-CBT | 8 | 41.00, 6.41 |  | 47.00, 9.96 |  | 45.00, 9.81 | 2, 38 | 4.18,  .023 | .180 | 2, 38 | .192,  .826 | .010 |
|  | sc-CBT | 13 | 37.85, 8.15 |  | 42.85, 9.05 |  | 43.38, 10.42 |  |  |  |  |  |  |

***Table S1.*** *Descriptive statistics (mean, standard deviation), and repeated measures ANOVA of primary outcomes as a function of 2 (CBT Group) by 5 (Time) design for PHQ-9, and 2 (CBT Group) by 3 (Time) design for QIDS and Q-LES-Q. Mauchly's test for all three questionnaires indicated that the assumption of sphericity was not violated.*

*Note.* i-CBT = internet-based cognitive behavioural therapy; sc-CBT = stepped care with electronic cognitive behavioural therapy; *N* = sample size; *M* = mean; *SD* = standard deviation; PHQ-9 = Patient Health Questionnaire; QIDS = Quick Inventory of Depressive Symptomatology; Q-LES-Q = Quality of Life Enjoyment and Satisfaction

***Table S2.*** *Post-hoc analysis using Bonferroni correction: Pairwise comparisons for PHQ-9, QIDS, Q-LES-Q of each timepoint.*

|  | **Session 0** | **Session 4** | **Session 7** | **Session 10** | **Session 13** | **Sessions 0 to 7** | **Sessions 7 to 13** | **Sessions 0 to 13** |
| --- | --- | --- | --- | --- | --- | --- | --- | --- |
|  | ***M, SE*** | ***M, SE*** | ***M, SE*** | ***M, SE*** | ***M, SE*** | ***Mean difference, SE, p***  ***95% Confidence Interval [Lower bound, Upper bound]*** | | |
| PHQ-9 | 15.946, 1.043 | 11.393,  1.159 | 10.973,  1.114 | 11.563,  1.347 | 10.589,  1.224 | 4.973, .973, <.001  [1.904, 8.042] | .804, 1.113, 1.000  [-2.707 , 4.314] | 5.357, 1.011, <.001  [2.169, 8.546] |
| QIDS | 13.967, 1.077 | N/A | 11.883,  1.361 | N/A | 9.850,  1.225 | 2.083, 1.171, .291 [-1.098, 5.265] | 2.033, 1.242, .371  [-1.341, 5.408] | 4.117, 1.235, .015  [.761, 7.473] |
| Q-LES-Q | 39.423, 1.698 |  | 44.923, 2.111 |  | 44.192, 2.292 | -5.500, 2.059, .045  [-10.906, -.094] | .731, 1.696, 1.000  [-3.723, 5.184] | -4.769, 2.384, .180  [-11.028, 1.489] |

*Note. M* = estimated marginal means; *SE* = standard error; PHQ-9 = Patient Health Questionnaire; QIDS = Quick Inventory of Depressive Symptomatology; Q-LES-Q = Quality of Life Enjoyment and Satisfaction

***Table S3.*** *Post-hoc analysis using Bonferroni correction: Pairwise comparisons for PHQ-9 of each timepoint. Statistics based on estimated marginal means.*

|  | | | | | **95% Confidence Interval for Difference** | |
| --- | --- | --- | --- | --- | --- | --- |
| **(I) Time** | **(J) Time** | **Mean Difference (I-J)** | **SE** | ***p*** | **Lower Bound** | **Upper Bound** |
| 1 | 2 | 4.554 | 1.043 | .002 | 1.264 | 7.843 |
|  | 3 | 4.973 | .973 | <.001 | 1.904 | 8.042 |
|  | 4 | 4.384 | 1.117 | .008 | .862 | 7.906 |
|  | 5 | 5.357 | 1.011 | <.001 | 2.169 | 8.546 |
| 2 | 3 | .420 | .829 | 1.000 | -2.196 | 3.035 |
|  | 4 | -.170 | 1.127 | 1.000 | -3.723 | 3.384 |
|  | 5 | .804 | 1.113 | 1.000 | -2.707 | 4.314 |
| 3 | 4 | -.589 | .816 | 1.000 | -3.161 | 1.983 |
|  | 5 | .384 | .942 | 1.000 | -2.587 | 3.355 |
| 4 | 5 | .973 | .747 | 1.000 | -1.383 | 3.329 |

* *Note: Time 1 is 0 weeks of treatment (baseline), time 2 is 4 weeks, time 3 is 7 weeks (mid-treatment), time 4 is 10 weeks, and time 5 is 13 weeks (post-treatment).*

***Table S4.*** *Post-hoc analysis using Bonferroni correction: Pairwise comparisons for QIDS of each timepoint. Statistics based on estimated marginal means.*

|  | | | | | **95% Confidence Interval for Difference** | |
| --- | --- | --- | --- | --- | --- | --- |
| **(I) Time** | **(J) Time** | **Mean Difference (I-J)** | **SE** | ***p*** | **Lower Bound** | **Upper Bound** |
| 1 | 2 | 2.083 | 1.171 | .291 | -1.098 | 5.265 |
|  | 3 | 4.117 | 1.235 | .015 | .761 | 7.473 |
| 2 | 3 | 2.033 | 1.242 | .371 | -1.341 | 5.408 |

* *Note: Time 1 is 0 weeks of treatment (baseline), time 3 is 7 weeks (mid-treatment), and time 5 is 13 weeks (post-treatment).*

***Table S5.*** *Post-hoc analysis using Bonferroni correction: Pairwise comparisons for Q-LES-Q of each timepoint. Statistics based on estimated marginal means.*

|  | | | | | **95% Confidence Interval for Difference** | |
| --- | --- | --- | --- | --- | --- | --- |
| **(I) Time** | **(J) Time** | **Mean Difference (I-J)** | **SE** | ***p*** | **Lower Bound** | **Upper Bound** |
| 1 | 2 | -5.500 | 2.059 | .045 | -10.906 | -.094 |
|  | 3 | -4.769 | 2.384 | .180 | -11.028 | 1.489 |
| 2 | 3 | .731 | 1.696 | 1.000 | -3.723 | 5.184 |

* *Note: Time 1 is 0 weeks of treatment (baseline), time 3 is 7 weeks (mid-treatment), and time 5 is 13 weeks (post-treatment).*
