# Supplementary figures and images for: Incorporating a Stepped Care Approach Into Internet-Based Cognitive Behavioral Therapy for Depression: Randomized Controlled Trial
Source: JMIR Ment Health. 2024 Feb 9;11:e51704. doi: 10.2196/51704 (PMC10891491; doi:10.2196/51704)

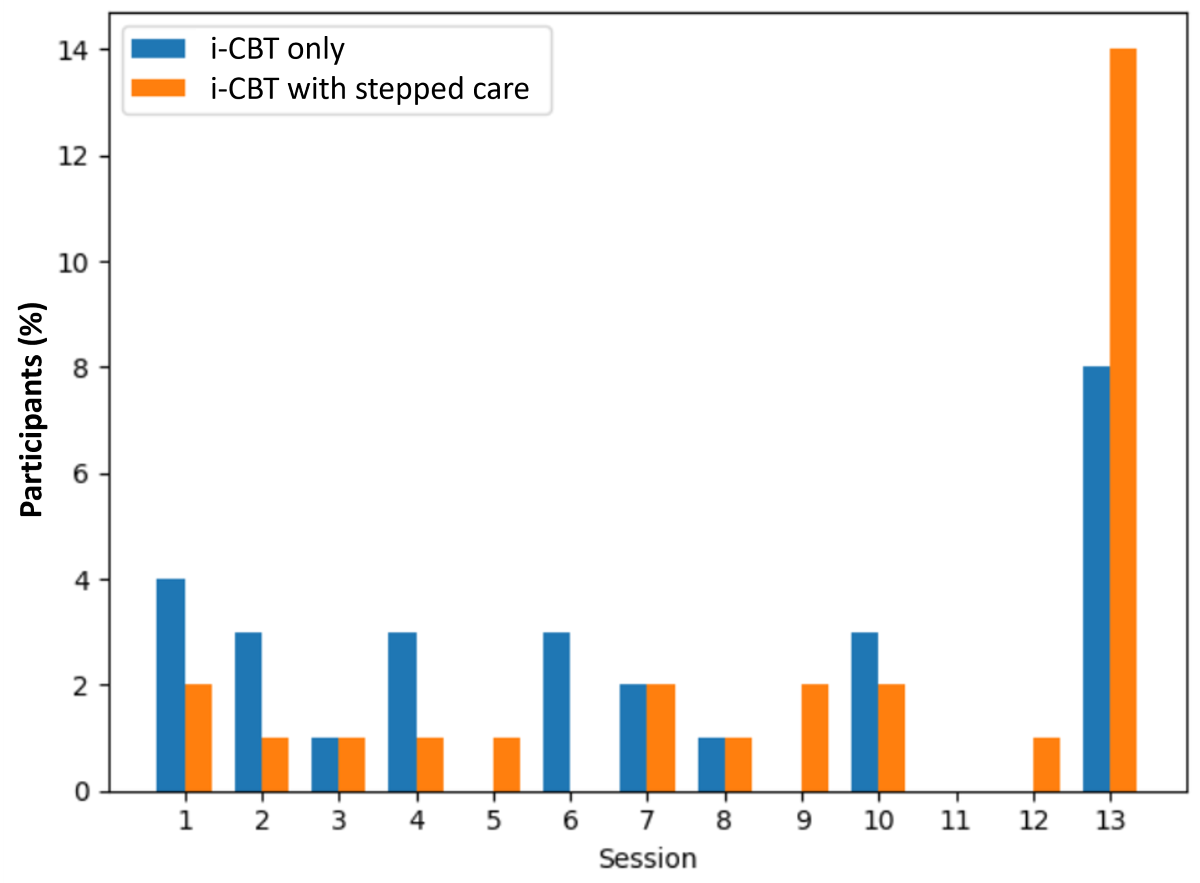

Supplement: Multimedia Appendix 5 [file mental_v11i1e51704_app5.png]
