# Supplementary material for: Incorporating a Stepped Care Approach Into Internet-Based Cognitive Behavioral Therapy for Depression: Randomized Controlled Trial
Source: JMIR Ment Health. 2024 Feb 9;11:e51704. doi: 10.2196/51704 (PMC10891491; doi:10.2196/51704)
Supplement: Multimedia Appendix 2 [file mental_v11i1e51704_app2.pdf]

ELECTRONIC **COGNITIVE BEHAVIOURAL THERAPY** (E-CBT)

Session 5

LET'S START

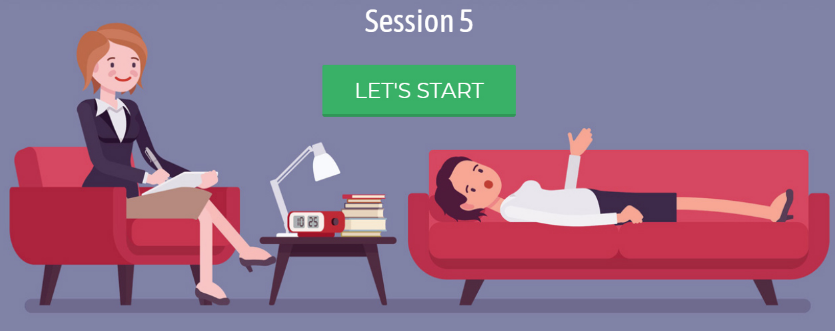

WELCOME BACK

Last week we talked about the connection between **Thoughts** and **Feelings**, **Behaviour**, **Physical Reactions** & **Environment**.

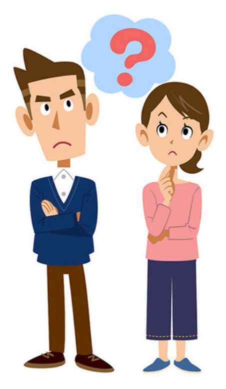

TODAY WE WILL TALK ABOUT "UNDERSTANDING AND RATING OUR FEELINGS" AND "THOUGHT RECORD"

In order to learn to control or change your feelings, it is helpful to be able to understand the feelings you are experiencing.

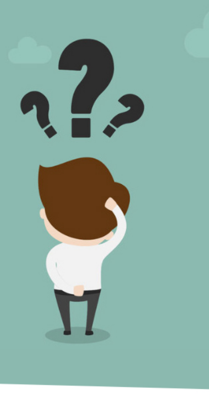

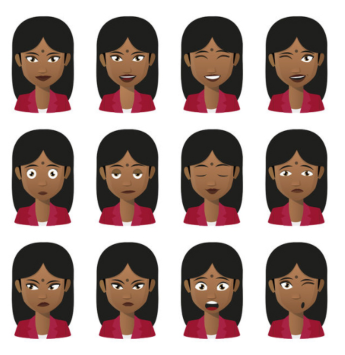

Different moods you might have during a day.

RATING MOODS

In addition to understanding your feelings, it is important to learn to rate the intensity of the feelings you experience.

There are some ways that can help you understand your feelings and behaviour and thoughts.

THESE 'WAYS' ARE SUMMARIZED IN A 7- COLUMN WORKSHEET CALLED A "THOUGHT RECORD"

THOUGHT RECORD

1. Situation	2. Feelings	3. Automatic Thoughts	4. Evidence that supports the hot thought	5. Evidence that doesn't support the hot thought	6. Alternative / Balanced Thoughts	7. Rate moods now

TODAY WE WILL TALK ABOUT THE FIRST 3 COLUMNS.

COLUMN 1: SITUATION

1. Use these 4 questions to fill out column 1.

- What happened?
- When did it happen?
- Who was involved?
- Where did it happen?

2. Be as specific as possible and limit the situation section to a specific time frame that does not exceed 30 minutes. For example: Friday, 3 pm.

Example: At 3:45 pm on Monday, at the office, I was told I didn't get the job I applied for.

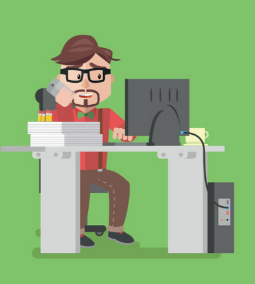

COLUMN 2: FEELINGS

Use these 2 questions to fill out column 2.

- What did you feel?
- Rate each feeling (0-100)

Example: Sad: 90%, Nervous: 80%, Humiliated: 60%

COLUMN 3: AUTOMATIC THOUGHTS

Use these 3 questions to fill out column 3.

- What was going through your mind just before you started to feel this way?
- Any other thoughts/images?
- Circle/highlight your hot thought

Example:

- Everyone thinks I am stupid
- I can never find a job
- **I will never succeed**
- Everybody is better than me

SUMMARY

- The "**Situation**" Column focuses on What, Where, When and Who.
- **Feelings** are identified in one word and rated for intensity on a 0-100 scale.
- The "**Automatic Thoughts** (images)" column describes thoughts, beliefs, concerns and meanings attached to the situation.

HOMEWORK #1

Fill the first 3 columns of a thought record for a situation that has happened in the past week.

1. Situation	2. Feelings	3. Automatic Thoughts

SUBMIT

HOMEWORK #2

Think of any recent problems you have experienced and describe each of these parts in the boxes below accordingly. You should submit your homework by the end of the weekend.

Worksheet

1. Situation / Environment changes

2. Physical Reactions

3. Feelings

4. Behaviours

5. Thoughts

SUBMIT

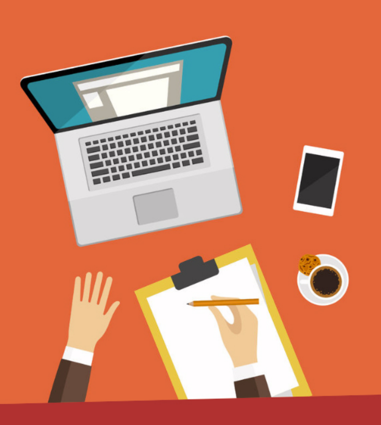

Don't forget to send your homework by the end of weekend.

You will receive feedback and next session's material next Wednesday.

THANK YOU FOR YOUR PARTICIPATION

Copyright © 2020 Company Name Ltd. | All Rights Reserved
